# Cerebrospinal fluid p-tau217 performs better than p-tau181 as a biomarker of Alzheimer’s disease

**DOI:** 10.1038/s41467-020-15436-0

**Published:** 2020-04-03

**Authors:** Shorena Janelidze, Erik Stomrud, Ruben Smith, Sebastian Palmqvist, Niklas Mattsson, David C. Airey, Nicholas K. Proctor, Xiyun Chai, Sergey Shcherbinin, John R. Sims, Gallen Triana-Baltzer, Clara Theunis, Randy Slemmon, Marc Mercken, Hartmuth Kolb, Jeffrey L. Dage, Oskar Hansson

**Affiliations:** 1 Clinical Memory Research Unit, Lund University, Sölvegatan 18, Lund, Sweden; 2 Department of Neurology, Skåne University Hospital, Entrégatan 7, 222 42 Lund, Sweden; 3 Wallenberg Center for Molecular Medicine, Lund University, Klinikgatan 32, 221 84 Lund, Sweden; 4 Eli Lilly and Company, Indianapolis, IN 46285 USA; 5 Neuroscience Biomarkers, Janssen Research & Development, 3210 Merryfield Row, San Diego, CA CA 92121 USA; 6 Janssen Pharmaceutical Companies of Johnson & Johnson, Turnhoutseweg 30, 2340 Beerse, Belgium; 7 Memory Clinic, Skåne University Hospital, Simrisbanvägen 14, 205 02 Malmö, Sweden

**Keywords:** Diagnostic markers, Alzheimer's disease

## Abstract

Cerebrospinal fluid (CSF) p-tau181 (tau phosphorylated at threonine 181) is an established biomarker of Alzheimer’s disease (AD), reflecting abnormal tau metabolism in the brain. Here we investigate the performance of CSF p-tau217 as a biomarker of AD in comparison to p-tau181. In the Swedish BioFINDER cohort (*n* = 194), p-tau217 shows stronger correlations with the tau positron emission tomography (PET) tracer [^18^F]flortaucipir, and more accurately identifies individuals with abnormally increased [^18^F]flortaucipir retention. Furthermore, longitudinal increases in p-tau217 are higher compared to p-tau181 and better correlate with [^18^F]flortaucipir uptake. P-tau217 correlates better than p-tau181 with CSF and PET measures of neocortical amyloid-β burden and more accurately distinguishes AD dementia from non-AD neurodegenerative disorders. Higher correlations between p-tau217 and [^18^F]flortaucipir are corroborated in an independent EXPEDITION3 trial cohort (*n* = 32). The main results are validated using a different p-tau217 immunoassay. These findings suggest that p-tau217 might be more useful than p-tau181 in the diagnostic work up of AD.

## Abstract (web summary)

Cerebrospinal fluid (CSF) p-tau181 (tau phosphorylated at threonine 181) is an established biomarker of Alzheimer’s disease (AD) reflecting abnormal tau metabolism in the AD brain. Here the authors demonstrate that CSF p-tau217 shows better performance as an AD biomarker than p-tau181.

## Introduction

Accumulation of intraneuronal neurofibrillary tangles (NFTs) containing paired helical filaments (PHFs) of the microtubule-associated protein tau is one of the defining neuropathological hallmarks of Alzheimer’s disease (AD)^1^. The tau protein has an N-terminal projection domain, a proline-rich region, a repeat region, and a C-terminal domain, with multiple potential phosphorylation sites along all regions^2^. Studies using preparations of PHFs and immunohistochemical staining of postmortem brain tissue with specific tau antibodies established that PHF tau is hyperphosphorylated^3,4^. High levels of p-tau and total tau (t-tau) have consistently been found in cerebrospinal fluid (CSF) of AD patients^5^. However, while CSF t-tau is considered a non-specific biomarker of neuronal injury, p-tau may reflect AD-related tau pathology in the brain^6^. The vast majority of CSF studies have used immunoassays detecting tau phosphorylated at threonine (Thr) 181 (p-tau181)^5^. During the last 2 decades, CSF p-tau181 together with total tau (t-tau) and amyloid-β 42 (Aβ42) have been extensively validated as biomarkers of AD and are currently widely used as diagnostic criteria in research studies, in clinical practice in some countries, and for patient selection in clinical trials^7–9^. CSF p-tau181 (alone or in combination with Aβ42) accurately differentiates AD from controls and predicts cognitive decline in preclinical and prodromal disease stages^10–12^. CSF p-tau181 levels are higher in AD compared with other tauopathies including frontotemporal dementia (FTD), progressive supranuclear palsy (PSP) and corticobasal degeneration (CBD) and, hence, CSF p-tau181 has also proven useful in differential diagnosis of dementia^10,13,14^.

Postmortem studies have shown that neocortical NFT load and hyperphosphorylated tau immunostaining both correlate with CSF p-tau, although the correlations have been modest^15,16^. In line with this observation, recent tau positron emission tomography (PET) investigations have found variable associations between imaging and CSF biomarkers of AD-related tau pathology^17–20^. The correlations appear to depend on clinical disease stage, since the associations between CSF p-tau181 and Tau PET measures were moderate in AD dementia, but weak or absent in cognitively unimpaired individuals and mild cognitive impairment (MCI) patients^19–21^. These finding are not surprising given that CSF concentrations of p-tau mirror abnormal tau metabolism (e.g. increased phosphorylation and release of soluble tau from damaged neurons) at a time of lumbar puncture whereas Tau PET is a measure of accumulation of insoluble paired helical filament tau over the disease course.

The role of tau phosphorylation at sites other that Thr181 has not been thoroughly investigated with one study demonstrating that p-tau181, p-tau199, and p-tau231 had similar diagnostic accuracies for AD^22^. Recently, the ratio of another p-tau isoform, p-tau217 (tau phosphorylated at Thr217), to t-tau, was found to correlate with Aβ deposition (measured using [^18^F]-AV45 PET) in AD^23^. Moreover, some preliminary evidence indicated that CSF p-tau217 might correlate more strongly with Tau PET measures than p-tau181 (conference abstract^24^). Extending these findings from relatively small cohorts, we analyzed CSF p-tau217 using a newly developed ELISA in a total of 226 individuals from two independent cohorts. We tested the associations of p-tau217 with Tau PET measures, CSF and PET measures of Aβ pathology and its diagnostic accuracy in comparison to p-tau181. A recent study using stable isotope labeling kinetics (SILK) method has suggested that that high CSF levels of tau proteins in AD may be due to amyloidosis-related increase in tau synthesis and active release from cells^23^. To assess whether increases in CSF p-tau levels in AD reflect hyperphosphorylation of specific residues in the brain or global changes in tau resulting from increased production and secretion, we compared p-tau217 and p-tau181 with t-tau, p-tau217/t-tau and p-tau181/t-tau. Both p-tau isoforms were measured using ELISA kits including the same detection antibody which limited the effects of reagent variability and allowed accurate comparison of biomarker performance. We found that CSF p-tau217 correlates stronger than p-tau181 with PET measures of tau and amyloid pathologies in AD and more accurately distinguishes AD dementia from non-AD neurodegenerative disorders. These results suggest potential benefit of implementing CSF p-tau217 as a biomarker of AD in clinical practice.

## Results

### Participants

We included 65 cognitively unimpaired controls (CU), 29 Aβ^+^ MCI due to AD (Aβ^+^ MCI), 43 AD dementia patients, and 57 patients with non-AD neurodegenerative disorders (Table 1). There were no differences in sex and education between different diagnostic groups, but patients with non-AD neurodegenerative disorders were somewhat younger on average than CU. The core CSF AD biomarkers (Aβ42, Aβ42/Aβ40, p-tau181) were increasingly abnormal in Aβ^+^ MCI and AD dementia.

**Table 1:** Demographic and clinical characteristics.

	Aβ^−^ CU *n* = 25	Aβ^+^ CU *n* = 40	Aβ^+^ MCI *n* = 29	AD dementia *n* = 43	Non-AD disorders^a^ *n* = 57	*P*-value
Age, years	75 (5)	74 (8)	72 (10)	72 (7)	70 (6)	0.002
Sex F/M, *n*	9/16	24/16	10/19	20/23	23/34	0.180
Education, years^b^	12 (4)	12 (4)	12 (4)	12 (4)	13 (4)	0.849
MMSE^b^	29 (1)	29 (1)	26 (3)	21 (5)	24 (6)	1.7 × 10^−19^
CSF Aβ42, pg/ml	686 (163)	398 (89)	337 (110)	299 (100)	526 (251)	6.0 × 10^−15^
CSF Aβ42/Aβ40	0.12 (0.03)	0.07 (0.02)	0.05 (0.01)	0.06 (0.02)	0.12 (0.04)	1.1 × 10^−20^
CSF p-tau217, pg/ml	94 (57)	247 (157)	684 (388)	812 (570)	135 (199)	1.9 × 10^−24^
CSF p-tau181, pg/ml	103 (44)	185 (91)	373 (167)	392 (221)	111 (89)	1.2 × 10^−21^
CSF t-tau, pg/ml	311 (92)	401 (131)	588 (163)	557 (226)	307 (142)	8.0 × 10^−15^
CSF p-tau217/t-tau	0.29 (0.10)	0.57 (0.22)	1.10 (0.40)	1.34 (0.47)	0.37 (0.25)	9.1 × 10^−27^
CSF p-tau181/t-tau	0.32 (0.05)	0.44 (0.10)	0.61 (0.14)	0.67 (0.14)	0.34 (0.11)	8.3 × 10^−25^
Braak I–II ROI	1.07 (0.09)	1.15 (0.19)	1.49 (0.29)	1.62 (0.22)	1.10 (0.16)	1.8 × 10^−20^
Braak III–IV ROI	1.15 (0.07)	1.19 (0.11)	1.64 (0.46)	1.94 (0.45)	1.19 (0.18)	4.9 × 10^−22^
Braak V–VI ROI	1.03 (0.06)	1.04 (0.06)	1.26 (0.27)	1.46 (0.36)	1.05 (0.11)	1.0 × 10^−16^

Data are shown as mean (SD) unless otherwise specified. Differences between the groups were tested using Kruskal–Wallis and chi-square (sex) tests.

*AD* Alzheimer’s disease, *bvFTD* behavioral-variant frontotemporal dementia, *CBS* corticobasal syndrome, *CSF* cerebrospinal fluid, *CU* cognitively unimpaired, *DLB* dementia with Lewy bodies, *F* female, *M* male, *MCI* mild cognitive impairment, *MMSE* Mini Mental State Examination, *PD* Parkinson’s disease, *PDD* Parkinson’s disease with dementia, *PSP* progressive supranuclear palsy, *SD* semantic dementia.

^a^Non-AD neurodegenerative disorders group included 10 PD, 17 PDD, 6 PSP, 7 DLB, 7 CBS, 4 SD, and 6 bvFTD patients.

^b^Education was missing for 1 Aβ^−^ CU and 2 FTD patients; MMSE was missing for 1 Aβ^−^ CU and 1 Aβ^+^ MCI, 3 AD dementia and 1 FTD patients; Tau PET data were missing for 1 Aβ^−^ CU, 1 Aβ^+^ CU, 1 Aβ^+^ MCI, 3 AD dementia, 1 PDD, 2 DLB, and 1 FTD patients.

### Group comparisons

**Fig. 1:**
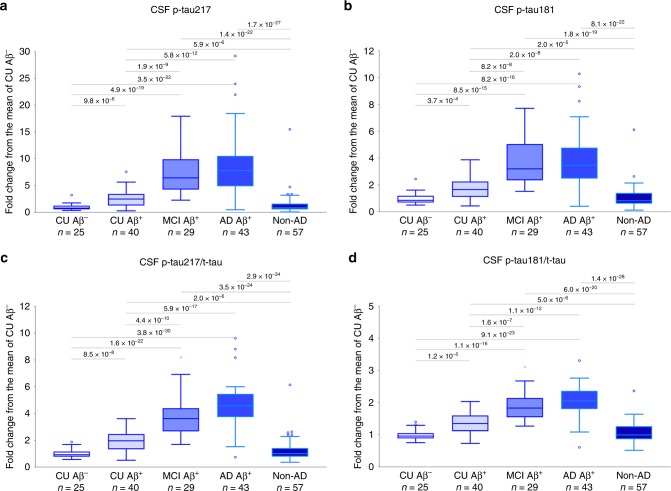
CSF p-tau in diagnostic groups. **a** CSF p-tau217, **b** CSF p-tau181, **c** CSF p-tau217/t-tau, and **d** CSF p-tau181/t-tau in CU Aβ^+^ (*n* = 25), CU Aβ^+^ (*n* = 40), MCI Aβ^+^ (*n* = 29), AD Aβ^+^ (*n* = 43) and non-AD neurodegenerative disorders (*n* = 57). Non-AD neurodegenerative disorders group included 10 PD, 17 PDD, 6 PSP, 7 DLB, 7 CBS, 4 SD, and 6 bvFTD patients. *P* values (unadjusted for multiple comparisons) are from univariate general linear models adjusted for age and sex; boxes show interquartile range, the horizontal lines are medians and the whiskers were plotted using Tukey method. Abbreviations: AD Alzheimer’s disease, bvFTD behavioral-variant frontotemporal dementia, CBS corticobasal syndrome, CSF cerebrospinal fluid, CU cognitively unimpaired controls, DLB dementia with Lewy bodies, MCI Mild Cognitive Impairment, PD Parkinson’s disease, PDD Parkinson’s disease with dementia, PSP progressive supranuclear palsy, SD semantic dementia.

CSF p-tau217 and p-tau181 were highly correlated (whole cohort Pearson *r* = 0.973, *p* = 8.8 × 10^−125^; CU Pearson *r* = 0.979, *p* = 1.8 × 10^−45^; Aβ^+^ MCI Pearson *r* = 0.972, *p* = 2.0 × 10^−18^; AD dementia Pearson *r* = 0.958, *p* = 8.6 × 10^−24^; non-AD neurodegenerative disorders Pearson *r* = 0.969, *p* = 3.3 × 10^−35^). Correlations with t-tau were stronger for p-tau181 than p-tau217 in the whole cohort and in different diagnostic groups (Supplementary Table 1). Both biomarkers as well as their ratios to t-tau were increased in all three Aβ^+^ groups compared to Aβ^−^ CU and non-AD groups (Fig. 1, Supplementary Figs. 1 and 2a). At the same time, the dynamic range was greater for p-tau217: the mean CSF concentration of p-tau217 was 7.3–8.6-fold higher in Aβ^+^ MCI and Aβ^+^ AD than in Aβ^−^ CU, whereas we only observed a 3.6–3.7-fold increase for p-tau181. The dynamic range was also greater for p-tau217/t-tau than p-tau181/t-tau (3.8–4.6-fold increase for p-tau217/t-tau vs 1.4–1.5-fold increase for p-tau181/t-tau). There were no differences in p-tau217, p-tau181 or t-tau levels between Aβ^+^ non-AD neurodegenerative disorders and Aβ^−^ non-AD neurodegenerative disorders (all *p* > 0.133).

### Associations with Tau PET

**Fig. 2:**
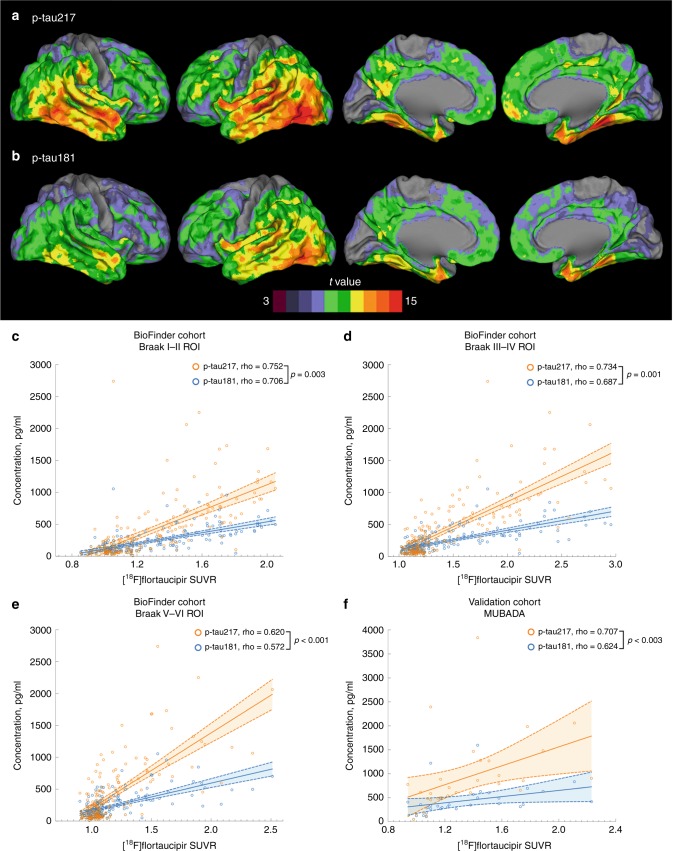
Associations between[^18^F]flortaucipir and p-tau. **a**, **b** BioFINDER cohort, voxel-wise regression analysis of p-tau217 (**a**) and p-tau181 (**b**) vs [^18^F]flortaucipir corrected for age. **c**–**e** BioFINDER cohort, associations between [^18^F]flortaucipir retention in a priori defined brain regions linked to tau pathology in AD and CSF p-tau217 and p-tau181. **f** Validation cohort, associations between [^18^F]flortaucipir MUBADA SUVR and CSF p-tau217 and p-tau181. In **c**–**f**, data are shown as Spearman correlation coefficients (rho), lines are linear regression lines with 95% CI (shaded area). Differences between the correlation coefficients were tested using estimated Spearman coefficients and method described in Rosner et al.^63^. Abbreviations: ROI region of interest, SUVR standardized uptake value ratio.

[^18^F]flortaucipir PET was performed in 184 study participants. First, we studied associations between the two p-tau isoforms and [^18^F]flortaucipir retention in three commonly used pre-defined brain regions including composites corresponding to Braak stage I–II, III–IV, and V–VI regions of interest (ROIs) (Supplementary Fig. 2b–d)^25^. We found that higher levels of p-tau217 and p-tau181 were both associated with increased [^18^F]flortaucipir retention in all three regions (Table 2). However, correlation coefficients were consistently higher for p-tau217 compared with p-tau181 and the differences between the coefficients were statistically significant (Fig. 2, Table 2 and Supplementary Fig. 3). Next, we studied the associations between CSF p-tau and [^18^F]flortaucipir in Aβ^−^ CU, Aβ^+^ CU, Aβ^+^ MCI and AD dementia cases, separately (Table 2). As expected, there were no correlations in Aβ^−^ CU cases. In Aβ^+^ CU cases, both p-tau variants correlated with [^18^F]flortaucipir in the early Braak I–II ROI, and this correlation was significantly stronger for p-tau217 than p-tau181. Further, in this group, p-tau217 correlated also with Braak III–IV ROI, which was not the case for p-tau181. In Aβ^+^ MCI p-tau217 correlated with all three Braak ROIs, but p-tau181 only correlated with Braak I–II ROI and to a significantly lower degree than p-tau217. Finally, in AD patients both p-tau isoforms correlated with later Braak III–IV and V–VI ROIs, and again the correlations for p-tau217 were significantly stronger than those for p-tau181 (Table 2). The correlation coefficients for CSF t-tau were lower across all regions and all groups (Table 2).

**Table 2:** Spearman correlations between CSF tau variants and regional [^18^F]flortaucipir retention.

	Braak I–II ROI	Braak III–IV ROI	Braak V–VI ROI
All (*n* = 184)			
p-tau181	**0.706** (**4.1** **×** **10**^−**29**^)	**0.687** (**4.8** **×** **10**^−**27**^)	**0.572** (**2.1** **×** **10**^−**17**^)
p-tau217	**0.752** (**8.6** **×** **10**^−**35**^)^a^	**0.734** (**1.9** **×** **10**^−**32**^)^b^	**0.620** (**5.8** **×** **10**^−**21**^)^c^
p-tau181/t-tau	**0.741** (**2.5** **×** **10**^−**33**^)	**0.752** (**9.1** **×** **10**^−**35**^)	**0.625** (**2.6** **×** **10**^−**21**^)
p-tau217/t-tau	**0.778** (**1.5** **×** **10**^−**38**^)^d^	**0.774** (**6.3** **×** **10**^−**38**^)^e^	**0.660** (**2.2** **×** **10**^−**24**^)^f^
t-tau	**0.599** (**2.9** **×** **10**^−**19**^)	**0.566** (**5.3** **×** **10**^−**17**^)	**0.464** (**3.4** **×** **10**^−**11**^)
Aβ^−^ CU (*n* = 24)			
p-tau181	0.142 (0.509)	−0.125 (0.560)	−0.247 (0.245)
p-tau217	0.277 (0.191)	−0.181 (0.398)	−0.338 (0.106)
p-tau181/t-tau	0.161 (0.453)	0.115 (0.593)	−0.032 (0.881)
p-tau217/t-tau	0.270 (0.201)	−0.153 (0.475)	−0.347 (0.010)
t-tau	0.052 (0.809)	−0.174 (0.416)	−0.250 (0.240)
Aβ^*+*^ CU (*n* = 39)			
p-tau181	**0.370** (**0.020**)	0.307 (0.058)	0.122 (0.458)
p-tau217	**0.456** (**0.004**)^g^	**0.363** (**0.023**)^h^	0.165 (0.314)
p-tau181/t-tau	**0.469** (**0.003**)	**0.387** (**0.015**)	0.202 (0.217)
p-tau217/t-tau	**0.556** (**2.3** **×** **10**^**−4**^)^i,k,l^	**0.433** (**0.006**)^j,m^	0.228 (0.163)
t-tau	0.263 (0.105)	0.202 (0.217)	0.048 (0.773)
Aβ^*+*^ MCI (*n* = 28)			
p-tau181	**0.550** (**0.002**)	0.324 (0.093)	0.302 (0.118)
p-tau217	**0.663** (**1.2** **×** **10**^**−4**^)^c^	**0.535** (**0.003**)^c^	**0.460** (**0.014**)^b^
p-tau181/t-tau	**0.534** (**0.002**)	**0.403** (**0.033**)	0.345 (0.072)
p-tau217/t-tau	**0.620** (**4.4** **×** **10**^−**4**^)	**0.599** (**0.001**)^c,o^	**0.472** (**0.011**)^n^
t-tau	**0.500** (**0.007**)	0.273 (0.160)	0.293 (0.130)
AD dementia (*n* = 40)			
p-tau181	0.300 (0.060)	**0.400** (**0.011**)	**0.358** (**0.023**)
p-tau217	0.288 (0.071)	**0.552** (**2.2** **×** **10**^−**4**^)^b^	**0.535** (**3.8** **×** **10**^−**4**^)^c^
p-tau181/t-tau	0.206 (0.202)	**0.399** (**0.011**)	**0.318** (**0.046**)
p-tau217/t-tau	0.238 (0.138)	**0.760** (**1.3** **×** **10**^−**8**^)^b,p,o^	**0.742** (**4.2** **×** **10**^−**8**^)^c,q,o^
t-tau	0.234 (0.146)	**0.340** (**0.032**)	**0.336** (**0.034**)

Data are Spearman correlation coefficients (*p*-value) with significant results shown in bold. Differences between the correlation coefficients were tested using estimated Spearman coefficients and method described in Rosner et al.^63^. Correlation coefficients were consistently lower for t-tau than for p-tau181 and therefore t-tau was excluded from the analysis.

*AD* Alzheimer’s disease, *CSF* cerebrospinal fluid, CU cognitively unimpaired, *MCI* mild cognitive impairment due to Alzheimer’s disease, *ROI* region of interest.

^a^*p* = 0.003 compared with p-tau181.

^b^*p* = 0.001 compared with p-tau181.

^c^*p* < 0.001 compared with p-tau181.

^d^*p* = 0.037 compared with p-tau181.

^e^*p* = 0.013 compared with p-tau181.

^f^*p* = 0.008 compared with p-tau181.

^g^*p* = 0.011 compared with p-tau181.

^h^*p* = 0.040 compared with p-tau181.

^i^*p* = 0.005 compared with p-tau181.

^j^*p* = 0.034 compared with p-tau181.

^k^*p* = 0.015 compared with p-tau217.

^l^*p* = 0.001 compared with p-tau181/t-tau.

^m^*p* = 0.045 compared with p-tau181/t-tau.

^n^*p* = 0.040 compared with p-tau181.

^o^*p* < 0.001 compared with p-tau181/t-tau.

^p^*p* = 0.015 compared with p-tau217.

^q^*p* = 0.002 compared with p-tau217.

Next we studied the ratios of p-tau to t-tau. We found that the correlation coefficients were significantly higher for p-tau217/t-tau ratio than for p-tau181, p-tau217 and p-tau181/t-tau in the earliest Braak I–II ROI in Aβ^+^ CU and the later III–IV and V–VI ROIs in AD dementia (Table 2). In the smaller Aβ^+^ MCI group, there were either significantly higher correlations or strong trends for higher correlations between [^18^F]flortaucipir retention and p-tau217/t-tau compared with p-tau181/t-tau (*p* = 0.055 Braak I–II ROI; *p* < 0.001 Braak III–IV ROI; *p* = 0.062 Braak V–VI ROI).

To be able to establish how well the two CSF p-tau isoforms could predict normal vs abnormal Tau PET scans, we dichotomized [^18^F]flortaucipir outcomes based on the previously established standardized uptake value ratio (SUVR) cutoffs of 1.3^26^. Compared to p-tau181, p-tau217 was a significantly more accurate predictor of abnormal Tau PET status in all three regions (Table 3). Given that CSF levels of tau may increase in Aβ^+^ individuals before Tau PET becomes abnormal^19,26^ we performed similar analysis excluding Aβ^+^/CSF p-tau^+^/Tau PET^−^ study participants. As expected, the AUC values increased for both p-tau217 (Braak I–II AUC 0.975, 95%CI 0.955–0.995; Braak III–IV AUC 0.951, 95%CI 0.919-0.983; Braak V–VI AUC 0.969, 95%CI 0.931–1.00) and p-tau 181 (Braak I–II AUC 0.957, 95%CI 0.924–0.989; Braak III–IV AUC 0.941, 95%CI 0.900–0.982; Braak V–VI AUC 0.958, 95%CI 0.897–1.00). While t-tau showed consistently lower AUCs than p-tau181 in all three regions, we observed significantly better performance of the p-tau217/t-tau ratio compared to p-tau181, p-tau217 and p-tau181/t-tau when identifying Tau PET status in Braak III–IV and V–VI ROIs (Table 3).

**Table 3:** ROC analysis of CSF tau variants for identifying abnormal [^18^F]flortaucipir status.

	AUC	Cut-off pg/ml	Sensitivity %	Specificity %	Youden’s J
Braak I–II ROI					
p-tau181	0.912 (0.869–0.955)	217.0	82	88	0.694
p-tau217	0.933 (0.898–0.968)^a^	287.8	88	87	0.741
p-tau181/t-tau	0.936 (0.897–0.974)	0.480	92	88	0.801
p-tau217/t-tau	0.947 (0.913–0.981)^b^	0.695	93	89	0.823
t-tau	0.845 (0.786–0.905)	443.6	76	81	0.576
Braak III–IV ROI					
p-tau181	0.900 (0.851–0.949)	224.7	82	86	0.674
p-tau217	0.933 (0.896–0.970)^c^	441.6	80	94	0.744
p-tau181/t-tau	0.933 (0.895–0.970)	0.542	85	91	0.755
p-tau217/t-tau	0.959 (0.930–0.988)^d,e,f^	0.890	86	95	0.813
t-tau	0.833 (0.769–0.897)	459.8	76	81	0.563
Braak V–VI ROI					
p-tau181	0.876 (0.805–0.947)	231.6	94	73	0.668
p-tau217	0.923 (0.874–0.973)^g^	582.9	87	88	0.747
p-tau181/t-tau	0.923 (0.884–0.961)	0.566	94	80	0.739
p-tau217/t-tau	0.972 (0.951–0.993)^h,i,j^	0.985	100	84	0.837
t-tau	0.813 (0.723–0.903)	473.5	81	73	0.532

Data are shown as AUC (95% CI). [^18^F]flortaucipir data was dichotomized based on the SUVR cutoff of 1.3^26^. AUC of two ROC curves were compared with DeLong test^64^. AUCs were consistently lower for t-tau than for p-tau181 and therefore t-tau was excluded from the analysis.

AUC area under the curve, CI confidence interval, CSF cerebrospinal fluid, ROC receiver operating characteristic, ROI region of interest.

^a^*p* = 0.010 compared with p-tau181.

^b^*p* = 0.042 compared with p-tau181.

^c^*p* = 1.9 × 10^−4^ compared with p-tau181.

^d^*p* = 0.002 compared with p-tau181.

^e^*p* = 0.019 compared with p-tau217.

^f^*p* = 6.2 × 10^−4^ compared with p-tau181/t-tau.

^g^*p* = 2.2 × 10^−4^ compared with p-tau181.

^h^*p* = 0.004 compared with p-tau181.

^i^*p* = 0.025 compared with p-tau217.

^j^*p* = 1.2 × 10^−4^ compared with p-tau181/t-tau.

### Associations with amyloid biomarkers

Increased levels of p-tau217 and p-tau181 were associated with decreased levels of CSF Aβ42 and with increased neocortical [^18^F]flutemetamol retention, and the correlations were stronger for p-tau217 and p-tau217/t-tau compared with p-tau181 (Supplementary Table 2). Furthermore, the AUCs for prediction of abnormal CSF Aβ42 and Aβ PET status were significantly higher for p-tau217 than p-tau181 (Table 4). The correlation coefficients and AUCs were lower for CSF t-tau and Tau PET measures than for p-tau181 (Table 4 and Supplementary Table 2).

**Table 4:** ROC analysis of CSF tau variants for identifying abnormal amyloid status.

	[^18^F]flutemetamol PET	CSF Aβ42
Cohort 1	*n* = 138	*n* = 184
p-tau181	0.890 (0.823–0.957)	0.799 (0.733–0.864)
p-tau217	0.910 (0.845–0.974)^a^	0.827 (0.766–0.888)^b^
p-tau181/t-tau	0.914 (0.848–0.979)	0.859 (0.804–0.915)^b^
p-tau217/t-tau	0.914 (0.844–0.984)	0.871 (0.818–0.923)^c,d^
t-tau	0.813 (0.736–0.891)	0.713 (0.638–0.789)
Braak I–II ROI	0.831 (0.758–0.904)	0.738 (0.669–0.808)
Braak III–IV ROI	0.793 (0.710–0.876)	0.732 (0.660–0.804)
Braak V–VI ROI	0.757 (0.672–0.842)	0.799 (0.733–0.864)
Cohort 2	*n* = 330	*n* = 350
p-tau181	0.915 (0.878–0.952)	0.677 (0.615–0.740)
p-tau217	0.949 (0.923–0.976)^e^	0.736 (0.679–0.793)^f^
p-tau181/t-tau	0.895 (0.856–0.935)^g^	0.778 (0.728–0.827)^h^
p-tau217/t-tau	0.956 (0.931–0.980)^i,j^	0.824 (0.779–0.869)^k,l,m^
t-tau	0.863 (0.819–0.907)	0.626 (0.562–0.690)
Braak I–II ROI	N/A	N/A
Braak III–IV ROI	N/A	N/A
Braak V–VI ROI	N/A	N/A

Data are shown as AUC (95% CI). [^18^F]flutemetamol PET and CSF Aβ42 cutoffs were defined as described in the Methods. AUC of two ROC curves were compared with DeLong test. AUC were consistently lower for t-tau than for p-tau181 and therefore t-tau was excluded from the analysis.

AUC area under the curve, CI confidence interval, CSF cerebrospinal fluid, ROC receiver operating characteristic, PET positron emission tomography, ROI region of interest.

^a^*p* = 0.042 compared with p-tau181.

^b^*p* = 0.001 compared with p-tau181.

^c^*p* = 3.4 × 10^−5^ compared with p-tau181.

^d^*p* = 2.6 × 10^−4^ compared with p-tau217.

^e^*p* = 1.4 × 10^−4^ compared with p-tau181.

^f^*p* = 5.0 × 10^−9^ compared with p-tau181.

^g^*p* = 2.8 × 10^−4^ compared with p-tau217.

^h^*p* = 2.5 × 10^−5^ compared with p-tau181.

^i^*p* = 0.004 compared with p-tau181.

^j^*p* = 3.4 × 10^−6^ compared with p-tau181/t-tau.

^k^*p* = 1.8 × 10^−12^ compared with p-tau181.

^l^*p* = 3.8 × 10^−9^ compared with p-tau217.

^m^*p* = 0.002 compared with p-tau181/t-tau.

We corroborated the CSF biomarker findings in a previously described cohort of CU and MCI patients (*n* = 330)^27^ from the Swedish BioFINDER study (Table 4 and Supplementary Table 2). In addition, in this larger cohort, the correlations with CSF Aβ42 were significantly stronger for CSF p-tau217/t-tau than for p-tau181, p-tau217 and p-tau181/t-tau (Supplementary Table 2), and p-tau217/t-tau was a significantly better predictor of abnormal CSF Aβ42 status than p-tau181, p-tau217 and p-tau181/t-tau (Table 4).

### Diagnostic accuracy of CSF p-tau217 and p-tau181

Next, we investigated diagnostic accuracies of CSF p-tau217 and p-tau181 in differentiating AD dementia (*n* = 43) from non-AD neurodegenerative disorders (*n* = 57). In ROC analysis, p-tau217 showed significantly higher AUC than p-tau181 (0.943 vs. 0.914, *p* = 0.026). When using Youden index derived cutoffs, the sensitivity of p-tau217 was 12% higher (91% vs. 79%), whereas specificity was 5% lower (91% vs. 96%) compared with p-tau181. However, for cutoffs with a fixed sensitivity of 91%, p-tau217 showed higher specificity than p-tau181 (91% vs. 75%). Also, the CSF p-tau217/t-tau performed significantly better than CSF p-tau181 when distinguishing AD dementia from non-AD neurodegenerative disorders (0.960 vs. 0.914, *p* = 0.034).

Further, we observed significantly higher AUCs for p-tau217 and p-tau217/t-tau compared with p-tau181 when separating Aβ^−^ CU from Aβ^+^ CU and Aβ^+^ CU from AD dementia (Supplementary Table 3). There were strong trends for better performance of both p-tau217 (*p* = 0.058) and p-tau217/t-tau (*p* = 0.076) when separating Aβ^+^ CU from Aβ^+^ MCI. The AUCs were lower for t-tau compared with p-tau181 in all ROC analysis (Supplementary Table 3).

### Associations of longitudinal CSF p-tau changes with Tau PET

**Fig. 3:**
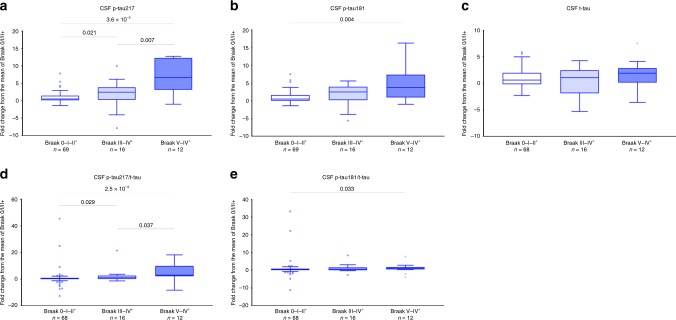
Longitudinal changes in CSF p-tau across the Braak ROI groups. Study participants were staged into different Braak ROI groups using [^18^F]flortaucipir PET. [^18^F]flortaucipir data was dichotomized based on the SUVR cutoff of 1.3^26^. Annual changes in CSF p-tau217 (**a**), p-tau181 (**b**), t-tau (**c**), p-tau217/t-tau (**d**), and p-tau181/t-tau (**e**) in the Braak 0-I–II (normal [^18^F]flortaucipir retention or abnormal [^18^F]flortaucipir retention limited to ROI I–II, *n* = 69), III–IV (abnormal [^18^F]flortaucipir retention in ROIs III–IV, *n* = 16) and V–VI (abnormal [^18^F]flortaucipir retention in ROI V–VI, *n* = 12) groups. *P* values (unadjusted for multiple comparisons) are from Mann–Whitney test; boxes show interquartile range, the horizontal lines are medians and the whiskers were plotted using Tukey method.

Ninety-seven study participants underwent an earlier LP before the main LP (which was closer to [^18^F]flortaucipir PET). In these individuals, we analyzed p-tau in two CSF samples and assessed associations between [^18^F]flortaucipir retention and the annual rate of change in the biomarker levels. The mean time between the two LPs was 3.4 years (SD = 1.4). We found a larger annualized increase in p-tau217 (7.0% [8.3%] of baseline, mean [SD]) than p-tau181 (4.6% [6.3%] of baseline, mean [SD]). Furthermore, longitudinal increases in the levels of p-tau217 and p-tau181 were associated with higher regional [^18^F]flortaucipir uptake. These correlations were significant only in the Aβ^+^ group and significantly higher for p-tau217 than for p-tau181 as well as for p-tau217/t-tau than for p-tau181/t-tau (Table 5). None of the correlations between longitudinal increase in CSF t-tau and [^18^F]flortaucipir uptake were significant (Table 5). In addition, for exploratory group-wise analyses, we compared longitudinal changes in the CSF p-tau levels between individuals that were [^18^F]flortaucipir negative or had an abnormal uptake only in the Braak I–II ROI (Braak 0–II^+^ group) vs. study participants with abnormal [^18^F]flortaucipir retention in the Braak III–IV and V–VI ROIs (Braak III–IV^+^ and V–VI^+^ groups) using the SUVR cutoff of 1.3^26^ (Fig. 3 and Supplementary Fig. 4). The rates of yearly increase in p-tau 217 and p-tau 217/t-tau were higher in both Braak III–IV^+^ and V–VI^+^ groups compared to 0/I–II^+^ group, but also in the Braak III–IV^+^ compared to V–VI^+^ groups. However, for p-tau181 and p-tau181/t-tau, the increases were only higher in the latest ROI V–VI^+^ group compared to the 0–II^+^ group with no other significant differences between the groups. We did not observe any differences between the groups in the rates of increase in CSF levels of t-tau (Fig. 3 and Supplementary Fig. 4).

**Table 5:** Spearman correlations between regional [^18^F]flortaucipir and longitudinal changes in CSF tau variants.

	Braak I–II ROI	Braak III–IV ROI	Braak V–VI ROI
All (*n* = 97)			
∆p-tau181	**0.381** (**1.2** **×** **10**^**−4**^)	**0.339** (**0.001**)	**0.201** (**0.048**)
∆p-tau217	**0.507** (**1.2** **×** **10**^−**7**^)^a^	**0.456** (**3.0** **×** **10**^−**6**^)^b^	**0.311** (**0.002**)^c^
∆p-tau181/t-tau	**0.284** (**0.005**)^d^	**0.270** (**0.008**)^e^	0.153 (0.136)
∆p-tau217/t-tau	**0.479** (**7.1** **×** **10**^−**7**^)^f^	**0.453** (**3.0** **×** **10**^−**6**^)^g^	**0.314** (**0.002**)^h^
∆t-tau	0.159 (0.120)	0.104 (0.309)	0.033 (0.746)
Aβ^+^ (*n* = 67)			
∆p-tau181	**0.376** (**0.002**)	**0.335** (**0.006**)	0.189 (0.126)
∆p-tau217	**0.519** (**7.0** **×** **10**^−**6**^)^i^	**0.505** (**1.3** **×** **10**^**−5**^)^j^	**0.369** (**0.002**)^j^
∆p-tau181/t-tau	0.238 (0.053)^k^	0.205 (0.096)^l^	0.086 (0.488)^m^
∆p-tau217/t-tau	**0.458** (**9.8** **×** **10**^**−5**^)^n^	**0.469** (**6.3** **×** **10**^**−5**^)^o^	**0.334** (**0.006**)^p^
∆t-tau	0.138 (0.266)	0.107 (0.387)	0.026 (0.833)
Aβ^−^ (*n* = 30)			
∆p-tau181	0.151 (0.426)	0.160 (0.398)	0.081 (0.670)
∆p-tau217	0.329 (0.075)	−0.044 (0.816)	−0.119 (0.533)
∆p-tau181/t-tau	0.092 (0.629)	0.130 (0.495)	0.013 (0.947)
∆p-tau217/t-tau	0.295 (0.113)	0.062 (0.745)	−0.097 (0.611)
∆t-tau	0.206 (0.274)	0.048 (0.800)	0.056 (0.768)

Delta (∆) p-tau181, ∆p-tau217, ∆p-tau181/t-tau, ∆p-tau217/t-tau and ∆t-tau represent yearly changes in the biomarker levels. Data are Spearman correlation coefficients (*p*-value) with significant results shown in bold. Differences between the correlation coefficients were tested using estimated Spearman coefficients and method described in Rosner et al.^63^. Correlations coefficients were consistently lower for t-tau than for p-tau181 and therefore t-tau was excluded from the analysis.

CSF cerebrospinal fluid, ROI region of interest.

^a^*p* = 0.001 compared with p-tau181.

^b^*p* = 0.012 compared with p-tau181.

^c^*p* = 0.011 compared with p-tau181.

^d^*p* = 0.028 compared with p-tau217.

^e^*p* = 0.031 compared with p-tau217.

^f^*p* = 0.004 compared with p-tau181/t-tau.

^g^*p* = 0.006 compared with p-tau181/t-tau.

^h^*p* = 0.019 compared with p-tau181/t-tau.

^i^*p* = 0.004 compared with p-tau181.

^j^*p* < 0.001 compared with p-tau181.

^k^*p* = 0.021 compared with p-tau217.

^l^*p* = 0.009 compared with p-tau217.

^m^*p* = 0.008 compared with p-tau217.

^n^*p* = 0.007 compared with p-tau181/t-tau.

^o^*p* = 0.001 compared with p-tau181/t-tau.

^p^*p* = 0.002 compared with p-tau181/t-tau.

### Validation in an independent cohort

To validate observations in the BioFINDER cohort, relationships between the two p-tau isoforms and [^18^F]flortaucipir signal were examined for amyloid positive mild AD participants of the EXPEDITION3 trial (*n* = 32, Supplementary Table 4). Although the population, PET acquisition, and processing parameters differ, we found close similarity between findings in the BioFINDER and EXPEDITION3 cohorts. Specifically, we found a positive association between levels of p-tau217 and p-tau181 and flortaucipir SUVR (Fig. 2f and Supplementary Fig. 3d). Moreover, the correlation coefficient was significantly higher for p-tau217 compared with p-tau181.

### Validation using different p-tau217 assay

In the BioFINDER subcohort (*n* = 178), we validated the results of the CSF p-tau217 measured using Eli Lilly assay by comparing another CSF p-tau217 assay developed by Janssen (p-tau217^J^) with p-tau181 assay from Eli Lilly. We found strong correlation between the p-tau217 data from the Janssen and Eli Lilly assays (Pearson *r* = 0.985, *p* = 8.0×10^−125^). Furthermore, in Aβ^+^ study participants (*n* = 132), p-tau217^J^ better correlated with [^18^F]flortaucipir SUVR in all three regions (Spearman rho 0.705–0.798, *p* = 4.2 × 10^−21^–3.0 × 10^−30^) than p-tau181 (Spearman rho 0.615–0.731, *p* = 4.3 × 10^−15^–2.5 × 10^−23^) (Supplementary Fig. 5). In ROC analysis, p-tau217^J^ was a more accurate predictor of abnormal Tau PET status than p-tau181 (Braak I–II AUC 0.945 vs. 0.918, *p* = 0.025; Braak III–IV AUC 0.944 vs. 0.904 *p* = 0.002; Braak V–VI AUC 0.926 vs. 0.869 *p* = 8.1 × 10^−4^). We also observed significantly higher AUC for p-tau217^J^ compared with p-tau181 (0.948 vs. 0.909 *p* = 0.012) when differentiating AD dementia (*n* = 42) from non-AD neurodegenerative disorders (*n* = 54).

## Discussion

Here we present evidence that CSF p-tau217 is a better biomarker of AD pathology than CSF p-tau181. Relative to the Aβ^−^ cognitively unimpaired group, increases in CSF concentrations of p-tau217 in prodromal AD and AD dementia were several folds higher compared with p-tau181. CSF p-tau217 showed higher correlations with [^18^F]flortaucipir and [^18^F]flutemetamol retention as well as with CSF Aβ42. Furthermore, this biomarker more accurately identified individuals with abnormally increased [^18^F]flortaucipir binding in different neocortical regions linked to tau pathology in AD. In addition, p-tau217 was significantly better than p-tau181 in distinguishing AD from a group of other neurodegenerative disorders. Longitudinal increases in p-tau217 were higher compared to p-tau181 and better correlated with [^18^F]flortaucipir retention in Aβ^+^ individuals. The main results were validated using different p-tau217 immunoassay. Finally, the findings of higher correlations between [^18^F]flortaucipir and p-tau217 compared with p-tau181 were validated in an independent cohort.

[^18^F]flortaucipir was developed to detect PHF-tau^28^ with several autoradiography investigations confirming its selective binding to tau aggregates containing PHF in AD^29–31^. In vivo retention of [^18^F]flortaucipir has been demonstrated to strongly correlate with the density of intrasomal tau tangles and tau-positive neurites in AD postmortem brain tissue^32^. Thr217 is one of roughly 40 different phosphorylation sites identified in PHF tau from Alzheimer brain^3^. Several antibodies specific for p-tau have been commonly used for immunostaining of human brain section to visualize and quantify tau pathology. One of these antibodies, AT100, binds to a conformational epitope that requires dual phosphorylation at Thr212 and serine (Ser) 214^33^. However, some data suggest that phosphorylation of nearby Thr217 is necessary for secondary phosphorylation of Thr212 and Ser 214 and that Thr217 is a part of A100 epitope^34,35^. Interestingly, AT270, an antibody that recognizes p-tau181, has been shown to stain fewer NFTs and neuropil threads in brain tissue from AD patients than AT100^36^, indicating that CSF p-tau217 might better reflect the pathological state of tau associated with PHF-tau formation. Accordingly, we found that correlations with [^18^F]flortaucipir in different brain regions were consistently higher for p-tau217 than p-tau181 and that [^18^F]flortaucipir retention was more related to longitudinal changes in p-tau217 than in p-tau181. In line with previous studies, the relationships between the CSF biomarkers and [^18^F]flortaucipir were disease stage dependent^17–21^. In the Aβ^+^ CU and Aβ^+^ MCI groups, higher levels of both CSF p-tau isoforms were associated with increased [^18^F]flortaucipir retention in the Braak I–II ROI (entorhinal cortex), one of earliest regions of tau pathology according to the Braak staging system^37,38^. In patients with AD dementia, these associations were significant only in more late Braak III–IV and V–VI ROIs. Importantly, we also observed significant correlations between p-tau217 and [^18^F]flortaucipir uptake in the Braak III–IV and V–VI ROIs in the Aβ^+^ MCI group where the correlations were not significant for p-tau181. Furthermore, p-tau217 was superior in detecting pathological [^18^F]flortaucipir status in all three regions. Thus, our results suggest that CSF p-tau217 better mirrors PET measures of tau pathology in different AD stages and especially in prodromal AD. Increase in CSF p-tau181 is considered to be specific for AD6. CSF levels of this biomarker have been shown to associate with Aβ PET^39^ and improve differentiation of AD from other tauopathies^10,13,14^. Providing further support for advantages of using p-tau217 over p-tau181 as a biomarker of AD, we found that CSF levels of p-tau217 correlated better with [^18^F]flutemetamol retention and distinguished more accurately AD from other dementias.

In agreement with previous data, the correlations with [^18^F]flortaucipir SUVR and measures of Aβ pathology as well as diagnostic performance of t-tau were lower compared with p-tau^10,19^. Further, in the majority of the cross-sectional data analysis in the present study, the p-tau217/t-tau ratio showed significantly higher correlation coefficients and AUCs than p-tau181/t-tau or p-tau 181. Taken together, these findings suggest that increases in CSF p-tau might reflect AD-related tau hyperphosphorylation rather than only increased production and secretion of tau from cells and that in AD changes in soluble tau metabolism affect to a larger extent phosphorylation at Thr217. This is in line with the study by Barthélemy et al.^40^, showing with mass spectrometry method increased tau phosphorylation rates in AD and better performance of pT217/T217 than pT181/T181 for discriminating AD vs non-AD^40^.

CSF p-tau181 is one of the core biomarkers incorporated into the NIA-AA Research Framework to define AD^6^. There is an ongoing effort to standardize protocols for determination of biomarker levels and to develop criteria for appropriate use of CSF testing in the diagnosis of AD with an intent of implementing CSF biomarkers into clinical practice^41,42^. The findings of the present study suggest that CSF p-tau217 is consistently more strongly related to the AD pathological process and might be more useful than p-tau181 in the diagnostic work up of AD. However, future validation studies in large independent cohorts and in routine clinical practice with samples collected and analyzed consecutively over long periods of time are required before adopting CSF p-tau217 as a clinically relevant biomarker. If the results are consistent, we suggest that p-tau217 replace p-tau181 in clinical routine practice.

## Methods

### Study population

The BioFINDER study was approved by the Regional Ethics Committee in Lund, Sweden (case number 2014/223), and all participants gave their written informed consent to participate in the study. Samples for the validation cohort were collected as part of a phase 3 clinical trial conducted in accordance with the Declaration of Helsinki for experiments involving human research. All participants gave their informed consent to participate in the study.

### BioFINDER cohort

Cognitively unimpaired controls (*n* = 65) and patients with MCI^43^ (*n* = 29), AD dementia^44^ (*n* = 43), PD^45^ (*n* = 10), PDD^46^ (*n* = 17), PSP^47^ (*n* = 6), DLB^48^ (*n* = 7), CBS^49^ (*n* = 7) and FTD^50,51^ (*n* = 10) were recruited from the Swedish BioFINDER study. All participants underwent a medical history, complete neurologic examination, neuropsychological testing and LP. Two LPs were performed in 97 study participants. One hundred eighty-four individuals (63 CU, 28 MCI, 70 AD, 10 PD, 16 PDD, 6 PSP, 5 DLB, 7 CBS, and 9 FTD) underwent [^18^F]flortaucipir PET. [^18^F]flutemetamol PET was performed in 139 study participants (61 CU, 24 MCI, 37 AD, 3 PSP, 3 DLB, 5 CBS, and 6 FTD). In this study, all MCI and AD patients as well as 39 (61%) CU were Aβ positive (according to either CSF Aβ42 or [^18^F]flutemetamol PET). In accordance with the research framework by the National Institute on Aging-Alzheimer’s Association CU included cognitively healthy individuals (*n* = 55) and participants with subjective cognitive decline (SCD, *n* = 10). The inclusion criteria for cognitively healthy elderly were (1) absence of cognitive symptoms as assessed by a physician with special interest in cognitive disorders, (2) age ≥60 years, (3) Mini–Mental State Examination (MMSE) 28−30 points at screening visit, (4) did not fulfill the criteria for MCI or any dementia disorder, and (5) fluency in Swedish. The exclusion criteria were (1) significant unstable systemic illness, such as terminal cancer, or organ failure that made it difficult to participate in the study, (2) current significant alcohol or substance misuse and (3) significant neurological or psychiatric illness. The inclusion criteria for patients with subjective cognitive decline (SCD) or MCI were (1) referred to a participating memory clinic because of cognitive complaints, (2) age 60–80 years, (3) did not fulfill the criteria for any dementia disorder and (4) fluency in Swedish. The exclusion criteria were (1) significant unstable systemic illness or organ failure, such as terminal cancer, that made it difficult to participate in the study, (2) current significant alcohol or substance misuse, and (3) cognitive impairment that without doubt could be explained by other specific non-neurodegenerative disorders, such as brain tumor or subdural hematoma. Following neuropsychological assessment including a test battery evaluating verbal ability, episodic memory function, visuospatial construction ability, and attention and executive functions, patients were classified as SCD or MCI. The characteristics of the study participants are given in Table 1.

### Validation cohort

We validated our observations using baseline biomarker storage samples and data acquired in EXPEDITION3 phase 3 trial with solanezumab (NCT01900665). EXPEDITION3 was a double-blind, placebo-controlled, phase 3 trial involving Aβ positive (shown by means of florbetapir PET or Aβ1-42 measurements in CSF) patients with mild dementia due to AD, defined as an MMSE score of 20–26^52^. Tau scanning with [^18^F]flortaucipir was performed at baseline, 40 weeks, and 80 weeks in a subset of subjects (approximately 10% of total subjects). CSF samples were collected during the trial in a subset of subjects and aliquots stored for future biomarker research. Overall, 32 participants had chosen to undergo both flortaucipir PET and lumbar puncture at baseline (before any treatment) and comprised the validation cohort and all 32 participants were included in the present study. The characteristics of those participants are given in Supplementary Table 4.

### CSF sampling and analysis

The procedure and analysis of CSF followed the Alzheimer’s Association Flow Chart for CSF biomarkers^10^. Lumbar CSF samples were collected at the 2 centers and analyzed according to a standardized protocol^53^. CSF Aβ42, Aβ40, and t-tau were measured with EUROIMMUN kits and according to the manufacturer’s recommendations.

### CSF p-tau181 and p-tau217 measurements (Lilly assays)

The p-tau181 and p-tau217 assays were designed for CSF analysis. Both assays were performed on a streptavidin small spot plate using the Meso Scale Discovery (MSD) platform (Meso Scale Discovery, Rockville, MD, USA). Anti-p-tau217 antibody IBA413 was used as a capture antibody in the p-tau217 assay whereas anti-p-tau181 antibody AT270 was used as a capture antibody in the p-tau181 assay. Antibodies were conjugated with Biotin (Thermo Scientific, catalog number: 21329) or SULFO-TAG (MSD, catalog number: R91AO-1). The assays were calibrated using a recombinant tau (4R2N) protein that was phosphorylated in vitro using a reaction with glycogen synthase kinase-3 and characterized by mass spectrometry. The samples were thawed on wet ice, briefly vortexed, and diluted 1:8 in Diluent 35 (MSD, catalog number: R50AE) with the addition of a heterophilic blocking reagent to a concentration of 200 µg/ml (Scantibodies Inc, catalog number: 3KC533). In order to perform the assays, MSD small-spot streptavidin coated plates (MSD, catalog number: L45SA) were blocked for 1 h at room temperature with 200 µl of 3% BSA in DPBS with 650 rpm shaking on a plate shaker. The plates were then washed three times with 200 µl of wash buffer (PBS + 0.05% Tween 20) and 25 µl of biotinylated capture antibody (AT270 for p-tau181 or IBA413 for p-tau217) at 1 µg/ml and 0.1 µg/ml respectively were added to the wells and incubated for 1 h at room temperature with 650 rpm shaking on a plate shaker. The plates were again washed three times with 200 µl of wash buffer and 50 µl of diluted calibrator or sample was added to each well and incubated for 2 h at room temperature with 650 rpm shaking on a plate shaker. The plates were then washed three times with 200 µl of wash buffer and 25 µl of SULFO-tagged LRL detection antibody was added at 3 µg/ml for the p-tau181 and at 0.5 µg/ml for the p-tau217 plates and incubated for 1 h at room temperature with 650 rpm shaking on a plate shaker. The plates were washed a final time with 200 µl of wash buffer and 150 µl of 2x MSD Read Buffer T with Surfactant (MSD, catalog number: R92TC) was added to each plate and read on the MSD SQ120 within 10 min of read buffer addition. Samples were analyzed in duplicates and the mean of duplicates were used in statistical analysis. The assay performances across all p-tau217 and p-tau181 plates are summarized in Supplementary Table 5 with a 10 pg/ml and 50 pg/ml QC buffer spike and high, medium, and low control CSF samples. Individual measurements all fell within 20% of the mean for QC and control samples. Selectivity and specificity were demonstrated across three experiments as described and shown in Supplementary Methods and Supplementary Fig. 6.

### CSF p-tau217^J^ assay

Validation of the CSF data using Lilly assays noted above was performed using a unique pair of assays developed at Janssen R&D (La Jolla, CA). CSF was measured in duplicate on Simoa HD-1 platform using p-tau217^J^ and t-Tau^J^ assays. Anti-p-tau217 antibody PT3 (Janssen R&D) was used as a capture antibody in the p-tau217^J^ assay, whereas anti-total tau antibody hT7 (ThermoFisher, Waltham, MA) was used as capture antibody in the t-Tau^J^ assay. Anti-total tau antibody PT82 (Janssen R&D) was used as detection antibody in both assays. All Simoa homebrew reagents and HD-1 instrument were obtained from Quanterix (Lexington, MA). The assays were calibrated using synthetic peptide containing the core epitope of the two sELISA antibodies separated by PEG4 linker (New England Peptide, Gardner, MA; MW = 4551 and 3619 g/mol for p-tau217^J^ and t-Tau^J^ assays, respectively). As this calibrant material is smaller than that used in the Lilly assays a direct comparison of absolute p-tau217 concentrations requires normalization.

### Tau and Aβ PET imaging and processing

BioFINDER cohort: [^18^F]flortaucipir and [^18^F]flutemetamol were synthesized at Skåne University Hospital, Lund, and PET scans were performed on a GE Discovery 690 PET scanner (Flortaucipir; General Electric Medical Systems, Chicago, IL) and a Philips Gemini TF 16 scanner (Flutemetamol; Philips Healthcare, Amsterdam, the Netherlands), respectively, as previously described^25,54^.

The mean injected dose of [^18^F]flortaucipir was ≈370MBq, and participants underwent a PET scan during the 80- to 100-min interval after injection. Images were motion corrected with the AFNI 3dvolreg, time averaged, and rigidly coregistered to the skull-stripped MRI scan. Standardized uptake value ratio (SUVR) images were created using inferior cerebellar gray matter as reference region^55^. FreeSurfer (version 5.3) parcellation of the T1-weighted MRI scan was applied to the PET data transformed to participants’ native T1 space to extract mean regional SUVR values for each participant in three predefined ROI corresponding to different image‐based stages of tau as described in Cho et al.^56^: the Braak I–II (entorhinal cortex), III–IV (parahippocampal gyrus, fusiform gyrus, amygdala, inferior temporal and middle temporal gyri) and V–VI (posterior cingulate gyrus, caudal anterior cingulate gyrus, rostral anterior cingulate gyrus, precuneus, inferior parietal lobule, superior parietal lobule, insula, supramarginal gyrus, lingual gyrus, superior temporal gyrus, medial orbitofrontal gyrus, rostral middle frontal gyrus, lateral orbitofrontal gyrus, caudal middle frontal gyrus, superior frontal gyrus, lateral occipital gyrus, precentral gyrus, postcentral gyrus and paracentral gyrus) ROIs. For voxel-wise analysis between [^18^F]flortaucipir and CSF p-tau the MR and PET images were transformed into Montreal Neurological Institute space (2 mm MNI152 MRI template) and voxel-wise correlations were made using multiple regressions adjusting for age in SPM12 (http://www.fil.ion.ucl.ac.uk/spm). Images were thresholded using family-wise error (FWE) correction at *p* < 0.01. The thresholded images were overlaid on a Population-Average, Landmark- and Surface-based (PALS) image^57^ using CARET v5.65 (Van Essen Lab; http://brainvis.wustl.edu). The images were not corrected for partial volume effects.

For [^18^F]flutemetamol, the mean injected dose was ≈185 MBq. PET images were acquired between 90 and 110 min after injection. The scanning and processing procedures have been described previously^53,58^. The motion corrected PET data was time-averaged and rigidly aligned with the anatomical MRI scan. Freesurfer (version 5.3) segmentations of the structural MR images were applied to extract SUVRs from a global neocortical region of interest. The weighted mean SUVR was calculated relative to a composite reference region (white matter, cerebellum and brainstem)^58,59^.

### Validation cohort

The Tau PET acquisitions were performed from 75 to 105 min (6 × 5 min frames) after injection of approximately 240 MBq of [^18^F]flortaucipir. Frames were aligned and averaged with an acquisition time-offset correction. Average 75–105 min image was spatially registered to the corresponding individual subject’s MRI space and then to the MRI template in Montreal Neurological Institute (MNI) stereotaxic space. Reference signal was parametrically derived in the white matter-based region to isolate non-specific signal (parametric estimate of reference signal intensity, PERSI^60^). The used weighted SUVR was designed by discriminant analysis that maximally separated diagnostic groups (multiblock barycentric discriminant analysis, MUBADA^61^).

### Statistical analysis

SPSS version 24 (IBM, Armonk, NY, US), SAS (9.4) and R version 3.4.3 (RStudio)^62^ were used for statistical analysis. Group differences in the biomarker levels were assessed with Mann–Whitney test or univariate general linear models (GLM) adjusting with age and sex as covariates. Log-transformed biomarker and PET measures were used in regression analyses. Correlations between CSF biomarkers and [^18^F]flortaucipir or [^18^F]flutemetamol SUVR were examined using Pearson or Spearman tests as indicated in the Results and Figure Legends. Differences between two dependent Spearman correlation coefficients were tested using methods described in Rosner et al.^63^ only when *p*-value for at least one correlation coefficient was <0.05. Diagnostic accuracies of CSF biomarkers were assessed using ROC curve analysis. AUC of two ROC curves were compared with DeLong test^64^. CSF Aβ42 (Youden’s cutoff < 519.9 pg/ml) and Aβ PET SUVR (Gaussian mixture modelling^65,66^ derived cutoff > 0.743) were used to define amyloid status. Study participants who underwent both lumbar puncture and Aβ PET imaging were considered Aβ positive if either CSF Aβ42 or Aβ PET measures were abnormal. We also dichotomized [^18^F]flortaucipir data based on the SUVR cutoff of 1.3^26^. Unadjusted two-sided *p* < 0.05 was considered statistically significant.

### Reporting summary

Further information on research design is available in the Nature Research Reporting Summary linked to this article.

## Supplementary information

Supplementary Information

Reporting Summary

## Data Availability

Anonymized data will be shared by request from a qualified academic investigator for the sole purpose of replicating procedures and results presented in the article and as long as data transfer is in agreement with EU legislation on the general data protection regulation and decisions by the Ethical Review Board of Sweden and Region Skåne, which should be regulated in a material transfer agreement.

## References

[1] Scheltens P (2016). Alzheimer’s disease. Lancet.

[2] Alonso AD, Beharry C, Corbo CP, Cohen LS (2016). Molecular mechanism of prion-like tau-induced neurodegeneration. Alzheimers Dement.

[3] Hanger DP, Anderton BH, Noble W (2009). Tau phosphorylation: the therapeutic challenge for neurodegenerative disease. Trends Mol. Med..

[4] Hernandez F, Avila J (2007). Tauopathies. Cell Mol. Life Sci..

[5] Olsson B (2016). CSF and blood biomarkers for the diagnosis of Alzheimer’s disease: a systematic review and meta-analysis. Lancet Neurol..

[6] Jack CR (2018). NIA-AA research framework: toward a biological definition of Alzheimer’s disease. Alzheimers Dement.

[7] Albert MS (2011). The diagnosis of mild cognitive impairment due to Alzheimer’s disease: recommendations from the National Institute on Aging-Alzheimer’s Association workgroups on diagnostic guidelines for Alzheimer’s disease. Alzheimers Dement..

[8] Dubois B (2014). Advancing research diagnostic criteria for Alzheimer’s disease: the IWG-2 criteria. Lancet Neurol..

[9] Molinuevo JL (2018). Current state of Alzheimer’s fluid biomarkers. Acta Neuropathol..

[10] Blennow K, Hampel H, Weiner M, Zetterberg H (2010). Cerebrospinal fluid and plasma biomarkers in Alzheimer disease. Nat. Rev. Neurol..

[11] Buchhave P (2012). Cerebrospinal fluid levels of beta-amyloid 1-42, but not of tau, are fully changed already 5 to 10 years before the onset of Alzheimer dementia. Arch. Gen. Psychiatry.

[12] Vos SJ (2013). Preclinical Alzheimer’s disease and its outcome: a longitudinal cohort study. Lancet Neurol..

[13] Lleo A (2018). A 2-Step cerebrospinal algorithm for the selection of frontotemporal lobar degeneration subtypes. JAMA Neurol..

[14] Schoonenboom NS (2012). Cerebrospinal fluid markers for differential dementia diagnosis in a large memory clinic cohort. Neurology.

[15] Buerger K (2006). CSF phosphorylated tau protein correlates with neocortical neurofibrillary pathology in Alzheimer’s disease. Brain.

[16] Tapiola T (2009). Cerebrospinal fluid {beta}-amyloid 42 and tau proteins as biomarkers of Alzheimer-type pathologic changes in the brain. Arch. Neurol..

[17] Brier MR (2016). Tau and Abeta imaging, CSF measures, and cognition in Alzheimer’s disease. Sci. Transl. Med..

[18] Chhatwal JP (2016). Temporal T807 binding correlates with CSF tau and phospho-tau in normal elderly. Neurology.

[19] Mattsson N (2017). (18)F-AV-1451 and CSF T-tau and P-tau as biomarkers in Alzheimer’s disease. EMBO Mol. Med..

[20] Mattsson N (2018). Comparing (18)F-AV-1451 with CSF t-tau and p-tau for diagnosis of Alzheimer disease. Neurology.

[21] Gordon BA (2016). The relationship between cerebrospinal fluid markers of Alzheimer pathology and positron emission tomography tau imaging. Brain.

[22] Hampel H (2004). Measurement of phosphorylated tau epitopes in the differential diagnosis of Alzheimer disease: a comparative cerebrospinal fluid study. Arch. Gen. Psychiatry.

[23] Sato C (2018). Tau kinetics in neurons and the human central nervous system. Neuron.

[24] Wildsmith KR (2018). TAU BURDEN MEASURED USING [18F]GTP1 CORRELATES WITH CSF TAU PHOSPHORYLATION AT SITES T217 AND T205 MORE CLOSELY THAN T181. Alzheimer’s Dement.: J. Alzheimer’s Assoc..

[25] Ossenkoppele R (2019). Associations between tau, Abeta, and cortical thickness with cognition in Alzheimer disease. Neurology.

[26] Ossenkoppele R (2018). Discriminative accuracy of [18F]flortaucipir positron emission tomography for Alzheimer disease vs other neurodegenerative disorders. JAMA.

[27] Janelidze S (2017). Concordance between different amyloid immunoassays and visual amyloid positron emission tomographic assessment. JAMA Neurol..

[28] Xia CF (2013). [(18)F]T807, a novel tau positron emission tomography imaging agent for Alzheimer’s disease. Alzheimers Dement.

[29] Lowe VJ (2016). An autoradiographic evaluation of AV-1451 Tau PET in dementia. Acta Neuropathol. Commun..

[30] Marquie, M. et al. Validating novel tau positron emission tomography tracer [F-18]-AV-1451 (T807) on postmortem brain tissue. *Ann. Neurol*. **78**, 787–800 (2015). 10.1002/ana.24517 PMC4900162 26344059

[31] Sander K (2016). Characterization of tau positron emission tomography tracer [(18)F]AV-1451 binding to postmortem tissue in Alzheimer’s disease, primary tauopathies, and other dementias. Alzheimers Dement.

[32] Smith Ruben, Wibom Moa, Pawlik Daria, Englund Elisabet, Hansson Oskar (2019). Correlation of In Vivo [18F]Flortaucipir With Postmortem Alzheimer Disease Tau Pathology. JAMA Neurology.

[33] Zheng-Fischhofer Q (1998). Sequential phosphorylation of Tau by glycogen synthase kinase-3beta and protein kinase A at Thr212 and Ser214 generates the Alzheimer-specific epitope of antibody AT100 and requires a paired-helical-filament-like conformation. Eur. J. Biochem..

[34] Hanger DP (2007). Novel phosphorylation sites in tau from Alzheimer brain support a role for casein kinase 1 in disease pathogenesis. J. Biol. Chem..

[35] Yoshida H, Goedert M (2006). Sequential phosphorylation of tau protein by cAMP-dependent protein kinase and SAPK4/p38delta or JNK2 in the presence of heparin generates the AT100 epitope. J. Neurochem..

[36] Spillantini MG, Crowther RA, Goedert M (1996). Comparison of the neurofibrillary pathology in Alzheimer’s disease and familial presenile dementia with tangles. Acta Neuropathol..

[37] Braak H, Alafuzoff I, Arzberger T, Kretzschmar H, Del Tredici K (2006). Staging of Alzheimer disease-associated neurofibrillary pathology using paraffin sections and immunocytochemistry. Acta Neuropathol..

[38] Braak H, Braak E (1991). Neuropathological stageing of Alzheimer-related changes. Acta Neuropathol..

[39] Blennow K, Mattsson N, Scholl M, Hansson O, Zetterberg H (2015). Amyloid biomarkers in Alzheimer’s disease. Trends Pharm. Sci..

[40] Barthélemy, N. R. et al. Tau hyperphosphorylation on T217 in cerebrospinal fluid is specifically associated to amyloid-β pathology. *bioRxiv*, 10.1101/226977 (2017).

[41] Hansson O (2018). The impact of preanalytical variables on measuring cerebrospinal fluid biomarkers for Alzheimer’s disease diagnosis: a review. Alzheimers Dement.

[42] Shaw LM (2018). Appropriate use criteria for lumbar puncture and cerebrospinal fluid testing in the diagnosis of Alzheimer’s disease. Alzheimers Dement.

[43] Petersen RC (2004). Mild cognitive impairment as a diagnostic entity. J. Intern. Med..

[44] McKhann GM (2011). The diagnosis of dementia due to Alzheimer’s disease: recommendations from the National Institute on Aging-Alzheimer’s Association workgroups on diagnostic guidelines for Alzheimer’s disease. Alzheimers Dement.

[45] Gelb DJ, Oliver E, Gilman S (1999). Diagnostic criteria for Parkinson disease. Arch. Neurol..

[46] Emre M (2007). Clinical diagnostic criteria for dementia associated with Parkinson’s disease. Mov. Disord..

[47] Litvan I (1996). Clinical research criteria for the diagnosis of progressive supranuclear palsy (Steele-Richardson-Olszewski syndrome): report of the NINDS-SPSP international workshop. Neurology.

[48] McKeith IG, Perry EK, Perry RH (1999). Report of the second dementia with Lewy body international workshop: diagnosis and treatment. Consortium on Dementia with Lewy Bodies. Neurology.

[49] Lang, A. E., Riley D. E., Bergeron C. Cortical-basal ganglionic degeneration. in *Neurodegenerative Diseases* (ed. Calne D. B.). (W.B. Saunders, 1994).

[50] Gorno-Tempini ML (2011). Classification of primary progressive aphasia and its variants. Neurology.

[51] Rascovsky K (2011). Sensitivity of revised diagnostic criteria for the behavioural variant of frontotemporal dementia. Brain.

[52] Honig LS (2018). Trial of Solanezumab for mild dementia due to Alzheimer’s disease. N. Engl. J. Med.

[53] Palmqvist S (2014). Accuracy of brain amyloid detection in clinical practice using cerebrospinal fluid beta-amyloid 42: a cross-validation study against amyloid positron emission tomography. JAMA Neurol..

[54] Hahn A (2017). Modeling strategies for quantification of in vivo (18)F-AV-1451 binding in patients with Tau pathology. J. Nucl. Med..

[55] Maass A (2017). Comparison of multiple tau-PET measures as biomarkers in aging and Alzheimer’s disease. Neuroimage.

[56] Cho H (2016). In vivo cortical spreading pattern of tau and amyloid in the Alzheimer disease spectrum. Ann. Neurol..

[57] Van Essen DC (2005). A population-average, landmark- and surface-based (PALS) atlas of human cerebral cortex. Neuroimage.

[58] Palmqvist S (2017). Earliest accumulation of beta-amyloid occurs within the default-mode network and concurrently affects brain connectivity. Nat. Commun..

[59] Palmqvist S (2019). Accurate risk estimation of beta-amyloid positivity to identify prodromal Alzheimer’s disease: cross-validation study of practical algorithms. Alzheimers Dement.

[60] Southekal S (2018). Flortaucipir F 18 quantitation using parametric estimation of reference signal intensity. J. Nucl. Med..

[61] Devous MD (2018). Test-retest reproducibility for the Tau PET imaging agent Flortaucipir F 18. J. Nucl. Med..

[62] Team, R. C. R.: *A Language and Environment for Statistical Computing*. (R Foundation for Statistical Computing, Vienna, 2014). http://www.R-project.org/.

[63] Rosner B, Wang W, Eliassen H, Hibert E (2015). Comparison of dependent pearson and spearman correlation coefficients with and without correction for measurement error. J. Biom. Biostat..

[64] Robin X (2011). pROC: an open-source package for R and S+ to analyze and compare ROC curves. BMC Bioinforma..

[65] Benaglia T, Chauveau D, Hunter DR, Young D (2009). S. mixtools: an R package for analyzing finite mixture models. J. Stat. Softw..

[66] Triana-Baltzer, G., et al. Development and validation of a high sensitivity assay for measuring p217+Tau in CSF. *J. Alzheimers Dis.* **62**, 737–744 (2018) 10.1002/dad2.12204 PMC8158165 34095436

