# Supplementary material for: Cerebrospinal fluid p-tau217 performs better than p-tau181 as a biomarker of Alzheimer’s disease
Source: Nat Commun. 2020 Apr 3;11:1683. doi: 10.1038/s41467-020-15436-0 (PMC7125218; doi:10.1038/s41467-020-15436-0)
Supplement: Supplementary file 1 — Supplementary Information [file 41467_2020_15436_MOESM1_ESM.pdf]

Supplementary Information

Cerebrospinal fluid p-tau217 performs better than p-tau181 as a biomarker of Alzheimer's disease

Janelidze et al.

Supplementary Methods

Selectivity for phosphorylated tau compared with non-phosphorylated tau in assay buffer was shown through comparison of the signal generated by the standard curve with the signal generated from levels of non-phosphorylated tau up to 1000 pg/mL. Non-phosphorylated tau (4R2N) did not generate significant signal at any levels tested, up to 1000 pg/mL (Supplementary Fig. 6a). In addition, the signal generated by the phosphorylated standard could be specifically blocked by the co-incubation of pT217 containing peptide (6 µg/mL) but not a pT181 containing peptide (6 µg/mL, Supplementary Fig. 6b). In order to confirm that the signal observed in human CSF sample is also specific, samples were analyzed with and without the coincubation of 6ug/mL of either pT217 or pT181 peptides. This resulted in complete blocking of the signal with the pT217 peptide but not with the pT181 peptide (Supplementary Fig. 6c).

Supplementary Table 1. Spearman correlations between CSF p-tau and t-tau.

	p-tau217	p-tau181	p-value p-tau217 vs. p-tau181
All (n=194)	0.896	0.936	<0.001
CU (n=65)	0.866	0.934	<0.001
$A\beta^+$ MCI (n=29)	0.896	0.943	<0.001
AD dementia (n=43)	0.923	0.947	0.047
Non-AD (n=57)	0.792	0.855	0.004

Data are Spearman correlation coefficients with all p value <0.001. Differences between the correlation coefficients were tested using estimated Spearman coefficients and method described in Rosner et al.[1].

AD, Alzheimer's disease; CSF, cerebrospinal fluid; CU, cognitively unimpaired; MCI, mild cognitive impairment.

Supplementary Table 2. Spearman correlations between CSF tau variants and amyloid biomarkers.

	[18F]flutemetamol PET	CSF Aβ42
**Cohort 1**	n=138	n=184
p-tau181	**0.600 (7.7x10^{-15})**	**-0.464 (3.3x10^{-11})**
p-tau217	**0.654 (3.5x10^{-18})[a]**	**-0.518 (5.2x10^{-14})[b]**
p-tau181/t-tau	**0.687 (1.4x10^{-20})**	**-0.583 (3.8x10^{-18})[b, d]**
p-tau217/t-tau	**0.700 (1.4x10^{-21})[c]**	**-0.612 (2.6x10^{-20})[b, e]**
t-tau	**0.449 (3.2x10^{-8})**	**-0.313 (1.6x10^{-5})**
Braak I-II ROI	**0.640 (3.1x10^{-17})**	**-0.387 (5.8x10^{-8})**
Braak III-IV ROI	**0.586 (4.3x10^{-14})**	**-0.410 (7.6x10^{-9})**
Braak V-VI ROI	**0.513 (1.3x10^{-10})**	**-0.330 (5.0x10^{-6})**
**Cohort 2**	n=330	n=350
p-tau181	**0.612 (2.4x10^{-35})**	**-0.280 (9.9x10^{-8})**
p-tau217	**0.666 (1.3x10^{-43})[f]**	**-0.402 (4.6x10^{-15})[b]**
p-tau181/t-tau	**0.662 (5.4x10^{-43})[g]**	**-0.488 (2.5x10^{-22})[b, h]**
p-tau217/t-tau	**0.726 (3.1x10^{-55})[b, e]**	**-0.587 (1.0x10^{-33})[b, e, i]**
t-tau	**0.485 (7.6x10^{-21})**	**-0.169 (0.001)**
Braak I-II ROI	N/A	N/A
Braak III-IV ROI	N/A	N/A
Braak V-VI ROI	N/A	N/A

Data are Spearman correlation coefficients (p-value) with significant results shown in bold. Differences between the correlation coefficients were tested using estimated Spearman coefficients and method described in Rosner et al.[1]. Correlation coefficients were consistently lower for t-tau and Tau PET measures than for p-tau181 and therefore these biomarkers were excluded from the analysis.
[a] p=0.004 compared with p-tau181; [b] p<0.001 compared with p-tau181; [c] p=0.032 compared with p-tau181; [d] p=0.005 compared with p-tau217; [e] p<0.001 compared with p-tau217; [f] p=0.004 compared with p-tau181/t-tau; [g] p=0.010 compared with p-tau181; [h] p=0.002 compared with p-tau217; [i] p<0.001 compared with p-tau181/t-tau.
N/A is used to indicate missing data.
CSF, cerebrospinal fluid; PET, positron emission tomography; ROI, region of interest.

Supplementary Table 3. ROC analysis of CSF tau variants for distinguishing different diagnostic groups.

	CU Aβ$^-$ vs CU Aβ$^+$	CU Aβ$^-$ vs Aβ+ MCI	CU Aβ$^-$ vs AD	CU Aβ$^-$ vs non-AD	CU Aβ$^+$ vs Aβ+ MCI	CU Aβ$^+$ vs AD	CU Aβ$^+$ vs non-AD	Aβ$^+$ MCI vs AD	Aβ$^+$ MCI vs non-AD	AD vs non-AD
p-tau181	**0.803 (0.695-0.911)**	**0.988 (0.964-1.00)**	**0.935 (0.869-1.00)**	0.463 (0.338-0.588)	**0.853 (0.766-0.939)**	**0.827 (0.736-0.917)**	**0.775 (0.681-0.868)**	0.513 (0.376-0.650)	**0.961 (0.922-1.00)**	**0.914 (0.848-0.980)**
p-tau217	**0.840** [a] **(0.742-0.938)**	**0.996 (0.986-1.00)**	**0.967 (0.921-1.00)**	0.515 (0.388-0.642)	**0.883 (0.805-0.960)**	**0.881**[c] **(0.806-0.957)**	**0.782 (0.688-0.875)**	0.564 (0.428-0.700)	**0.966 (0.929-1.00)**	**0.943** [g] **(0.892-0.994)**
p-tau181/t-tau	**0.843 (0.747-0.939)**	**0.990 (0.974-1.00)**	**0.972 (0.926-1.00)**	0.534 (0.411-0.657)	**0.864 (0.779-0.948)**	**0.925** [d] **(0.862-0.988)**	**0.758 (0.662-0.853)**	**0.649 (0.517-0.780)**	**0.947 (0.902-0.992)**	**0.954 (0.902-1.00)**
p-tau217/t-tau	**0.868** [b] **(0.782-0.954)**	**0.999 (0.994-1.000)**	**0.980 (0.943-1.00)**	0.580 (0.455-0.704)	**0.907 (0.837-0.977)**	**0.950** [e, f] **(0.895-1.00)**	**0.770 (0.674-0.866)**	**0.691 (0.566-0.817)**	**0.964 (0.925-1.00)**	**0.960** [h] **(0.913-1.00)**
t-tau	**0.711 (0.585-0.837)**	**0.948 (0.894-1.00)**	**0.845 (0.751-0.939)**	0.452 (0.323-0.581)	**0.823 (0.728-0.919)**	**0.725 (0.615-0.836)**	**0.715 (0.614-0.817)**	0.452 (0.320-0.585)	**0.916 (0.858-0.974)**	**0.830 (0.744-0.917)**

Data are shown as AUC (95% CI) with significant results shown in bold. For AUC values >0.800, AUCs of two ROC curves were compared with DeLong test[2]. AUCs were consistently lower for t-tau than for p-tau181 and therefore t-tau was excluded from the analysis. [a] p=0.022 compared with p-tau181; [b] p=0.046 compared with p-tau181; [c] p=8.1x10^{-05} compared with p-tau181; [d] p=0.005 compared with p-tau181; [e] p=0.001 compared with p-tau181; [f] p=0.013 compared with p-tau217; [g] p=0.026 compared with p-tau181; [h] p=0.034 compared with p-tau181.

AD, Alzheimer's disease; AUC, area under the curve; CI, confidence interval; CU, cognitively unimpaired; MCI, mild cognitive impairment; ROC, Receiver Operating Characteristic.

Supplementary Table 4. Demographic and clinical characteristics of the validation cohort.

	Amyloid Positive Mild AD<br>n=32
Age, *years*	73 (8)
Sex F/M, *n*	13/19
Education, *years*	15 (2)
MMSE	23 (2)
p-tau217, *pg/ml*	918 (773)
p-tau181, *pg/ml*	442 (313)

Data are shown as mean (SD) unless otherwise specified.
AD, Alzheimer's disease dementia; F, female; M, male; MMSE, Mini Mental State Examination.

Supplementary Table 5. Performance of the p-tau217 and p-tau181 assays.

Sample	Mean concentration, pg/ml	SD	N	% CV
**p-tau217**				
10 pg/mL QC	9.97	0.59	22	5.95
50 pg/mL QC	44.31	2.06	22	4.66
High Control Sample	737.99	40.96	22	5.55
Medium Control Sample	328.72	17.81	22	5.42
Low Control Sample	59.69	4.93	22	8.26
**p-tau181**				
10 pg/mL QC	9.49	0.24	22	2.53
50 pg/mL QC	45.84	1.05	22	2.29
High Control Sample	379.61	21.25	22	5.60
Medium Control Sample	190.27	7.29	22	3.83
Low Control Sample	71.19	3.92	22	5.51

CV, coefficient of variation; QC, quality control; SD, standard deviation.

Supplementary Figure 1

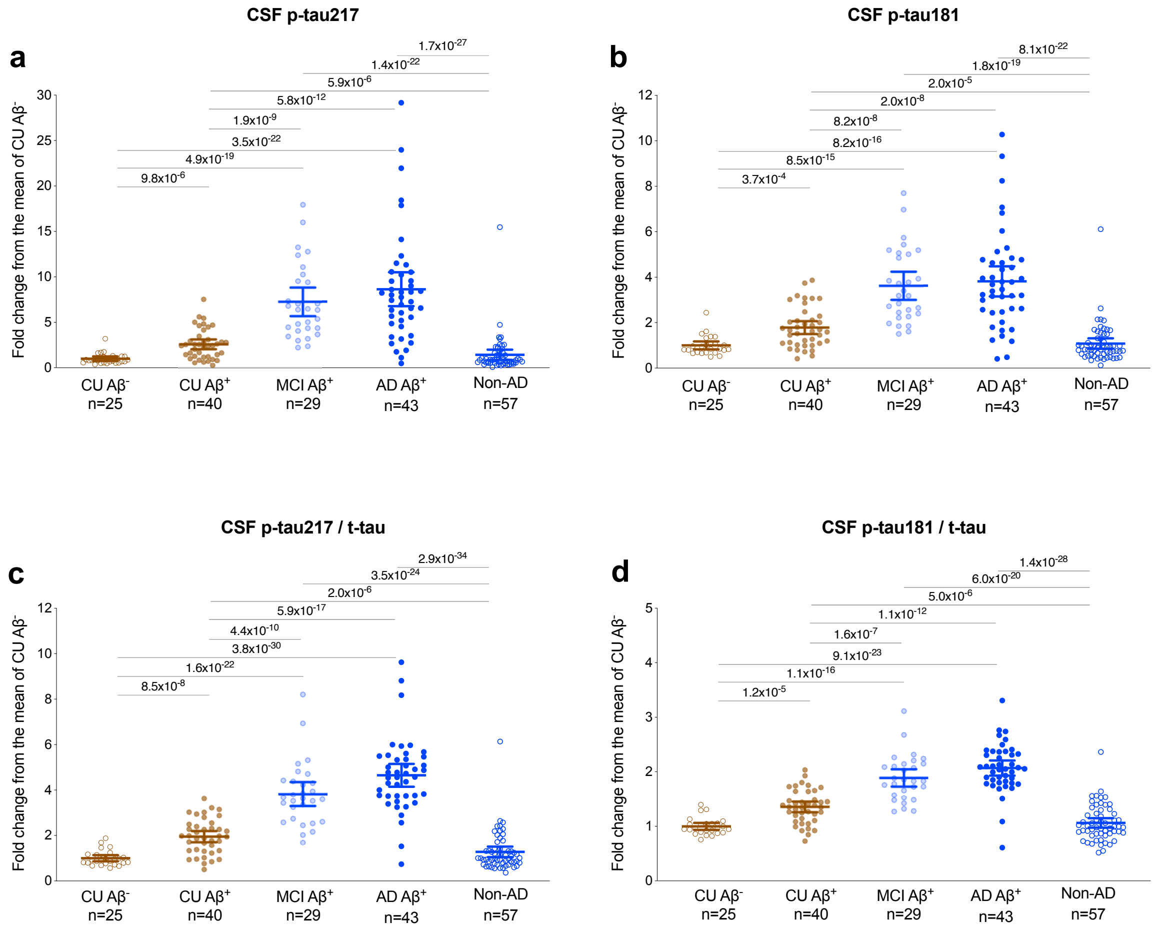

Scatter plots of CSF p-tau in diagnostic groups. CSF p-tau in CU Aβ+ (n=40), MCI Aβ+ (n=29), AD Aβ+ (n=43) and non-AD neurodegenerative disorders (n=57). (**a**) CSF p-tau217, scatter plot for Figure 1a; (**b**) CSF p-tau181, scatter plot for Figure 1b; (**c**) CSF p-tau217/t-tau, scatter plot for Figure 1c; and (**d**) CSF p-tau181/t-tau in CU Aβ+ (n=25), scatter plot for Figure 1d. P values (unadjusted for multiple comparisons) are from univariate general linear models adjusted for age and sex; solid horizontal lines represent mean and error bars correspond to 95% confidence interval.

Supplementary Figure 2

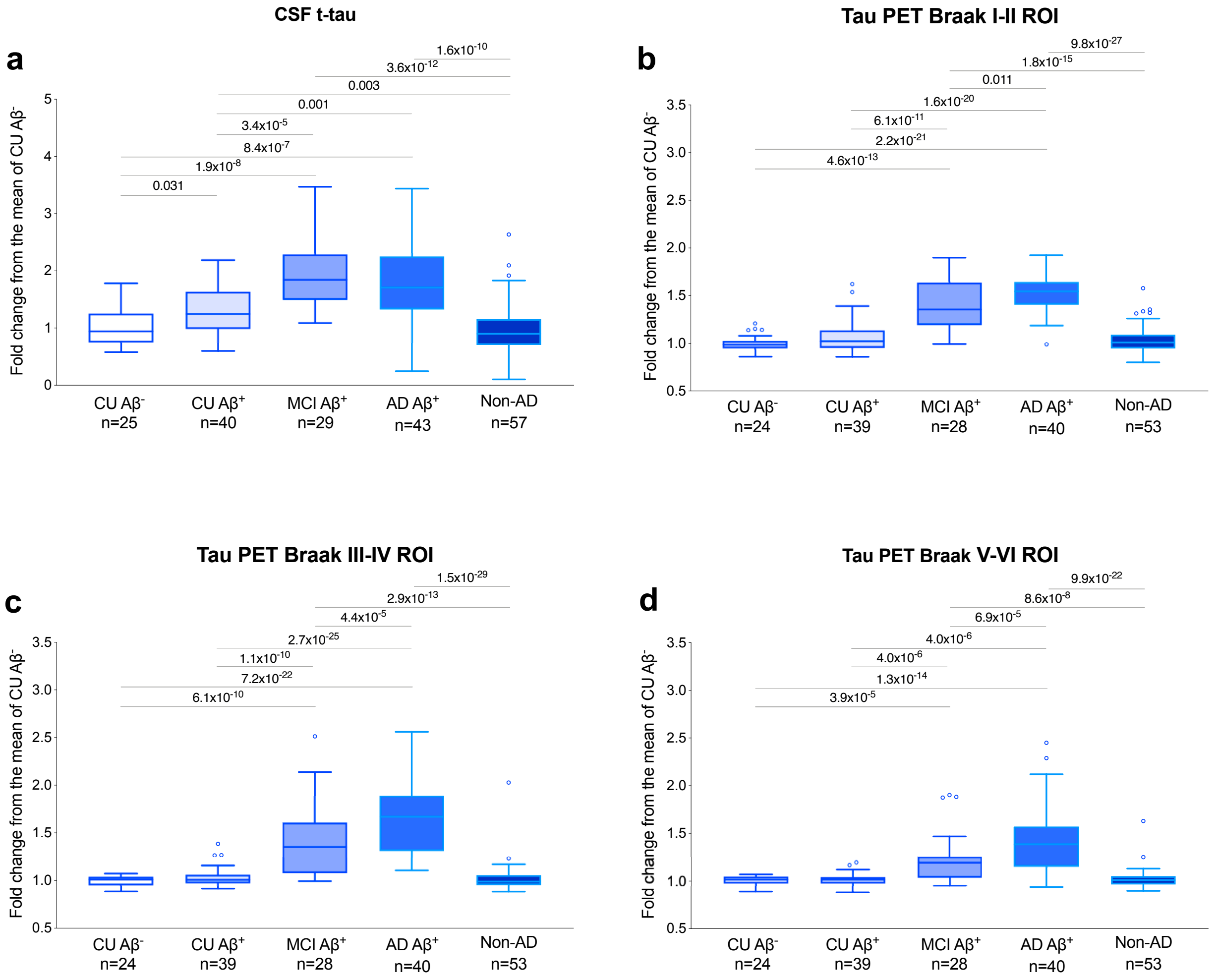

CSF t-tau and [18F]flortaucipir in diagnostic groups. CSF t-tau (**a**) and [18F]flortaucipir retention in Braak I-II (**b**), III-IV (**c**) and V-VI (**d**) ROI in CU Aβ+ (n=25 for t-tau, n=24 for Tau PET), CU Aβ+ (n=40 for t-tau, n=39 for Tau PET), MCI Aβ+ (n=29 for t-tau, n=28 for Tau PET), AD Aβ+ (n=43 for t-tau, n=40 for Tau PET) and non-AD neurodegenerative disorders (n=57 for t-tau, n=53 for Tau PET). Non-AD neurodegenerative disorders group included 10 PD, 17 PDD, 6 PSP, 7 DLB, 7 CBS, 4 SD and 6 bvFTD patients. P values (unadjusted for multiple comparisons) are from univariate general linear models adjusted for age and sex; boxes show interquartile range, the horizontal lines are medians and the whiskers were plotted using Tukey method. Data in partially overlapping cohort have been previously published[3] and are shown here for comparison. Abbreviations: AD, Alzheimer's disease; bvFTD, behavioral-variant frontotemporal dementia; CBS, corticobasal syndrome; CSF, cerebrospinal fluid; CU, cognitively unimpaired controls; DLB, dementia with Lewy bodies; MCI, Mild Cognitive Impairment; PD, Parkinson's disease; PDD, Parkinson's disease with dementia; PSP, progressive supranuclear palsy; SD semantic dementia.

Supplementary Figure 3

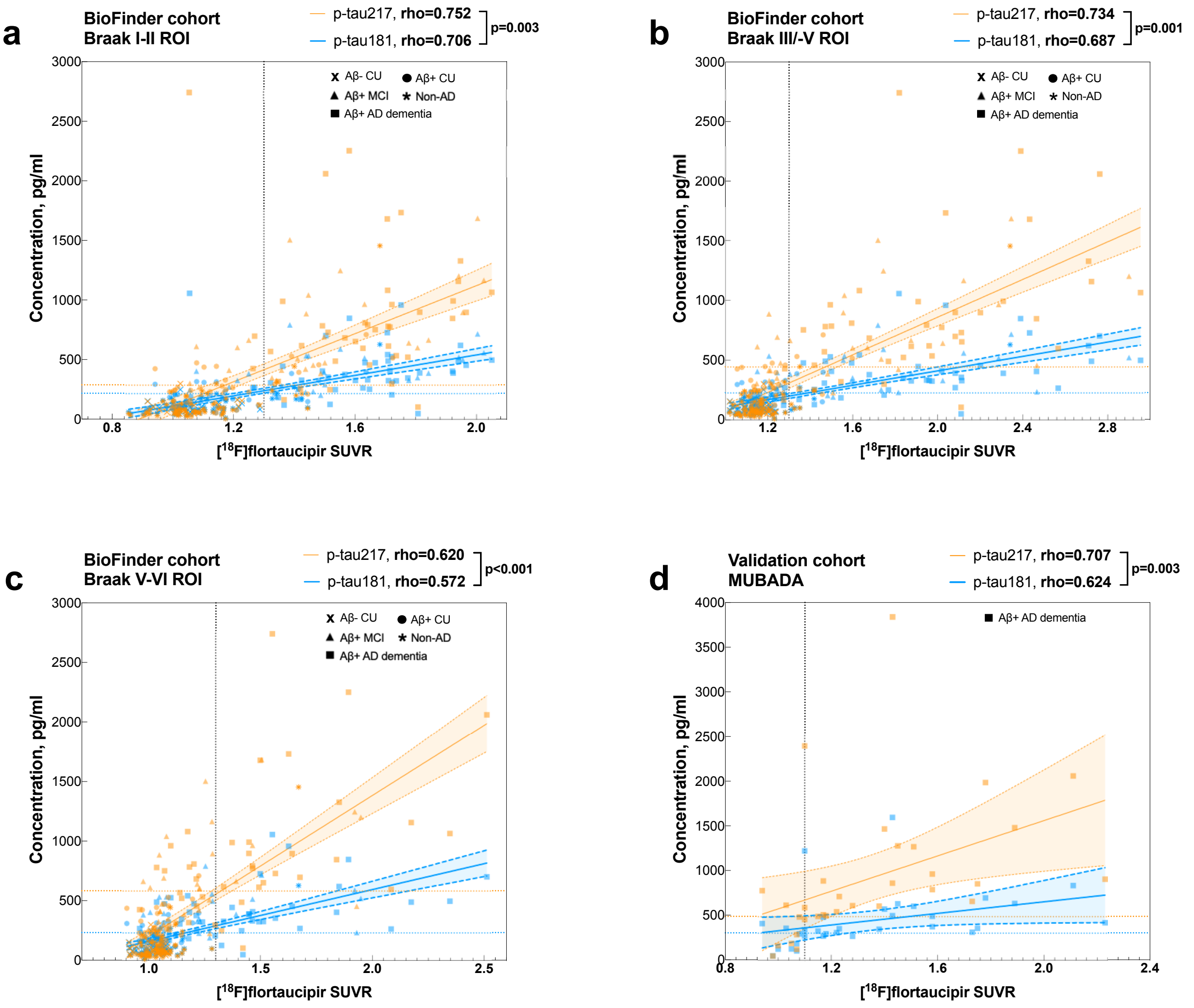

Associations between[18F]flortaucipir and p-tau. (**a-c**) BioFINDER cohort, associations between [^{18}F]flortaucipir retention in *a priori* defined brain regions linked to tau pathology in AD and CSF p-tau217 and p-tau181. (**d**) Validation cohort, associations between[^{18}F]flortaucipir MUBADA SUVR and CSF p-tau217 and p-tau181. Data are shown as Spearman correlation coefficients (rho); lines are linear regression lines with 95% CI (shaded area). Dotted lines indicate [^{18}F]flortaucipir SUVR cutoffs and Youden's index cutoffs for CSF p-tau217 and p-tau181. Differences between the correlation coefficients were tested using estimated Spearman coefficients and method described in Rosner et al.[1]. Abbreviations: ROI, region of interest; SUVR, standardized uptake value ratio.

Supplementary Figure 4

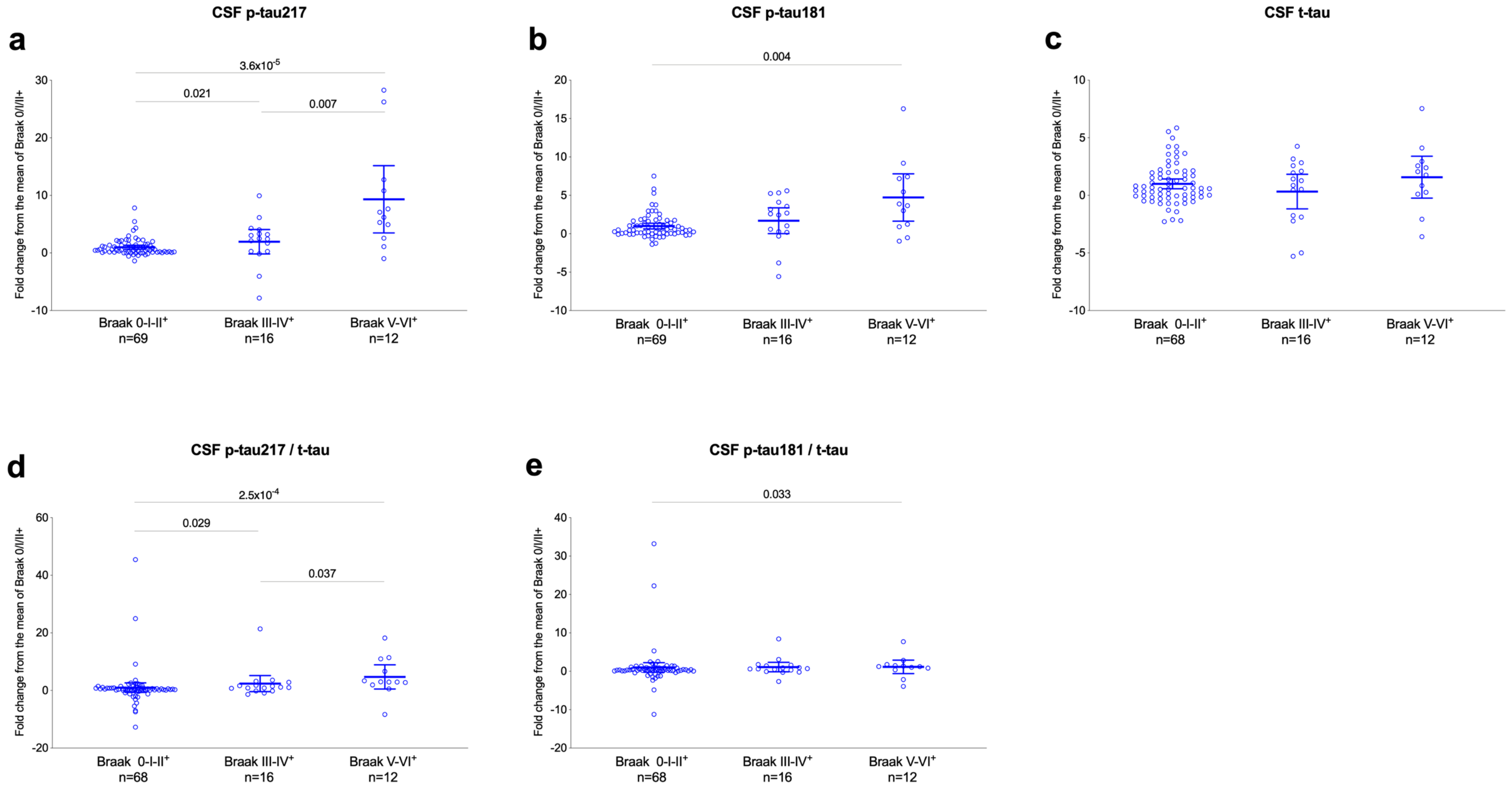

Scatter plots of longitudinal changes in CSF p-tau. Study participants were staged into different Braak ROI groups using [^{18}F]flortaucipir PET. [^{18}F]flortaucipir data was dichotomized based on the SUVR cutoff of 1.326. Annual changes in CSF p-tau217 (**a,** scatter plot for Figure 3a), p-tau181 (**b,** scatter plot for Figure 3b), t-tau (**c,** scatter plot for Figure 3c), p-tau217/t-tau (**d,** scatter plot for Figure 3d), and p-tau181/t-tau (**e,** scatter plot for Figure 3e) in the Braak 0-I-II (normal [^{18}F]flortaucipir retention or abnormal [^{18}F]flortaucipir retention limited to ROI I-II, n=69), III-IV (abnormal [^{18}F]flortaucipir retention in ROIs III-IV, n=16) and V-VI (abnormal [^{18}F]flortaucipir retention in ROI V-VI, n=12) groups. P values (unadjusted for multiple comparisons) are from Mann-Whitney test; solid horizontal lines represent mean and error bars correspond to 95% confidence interval.

Supplementary Figure 5

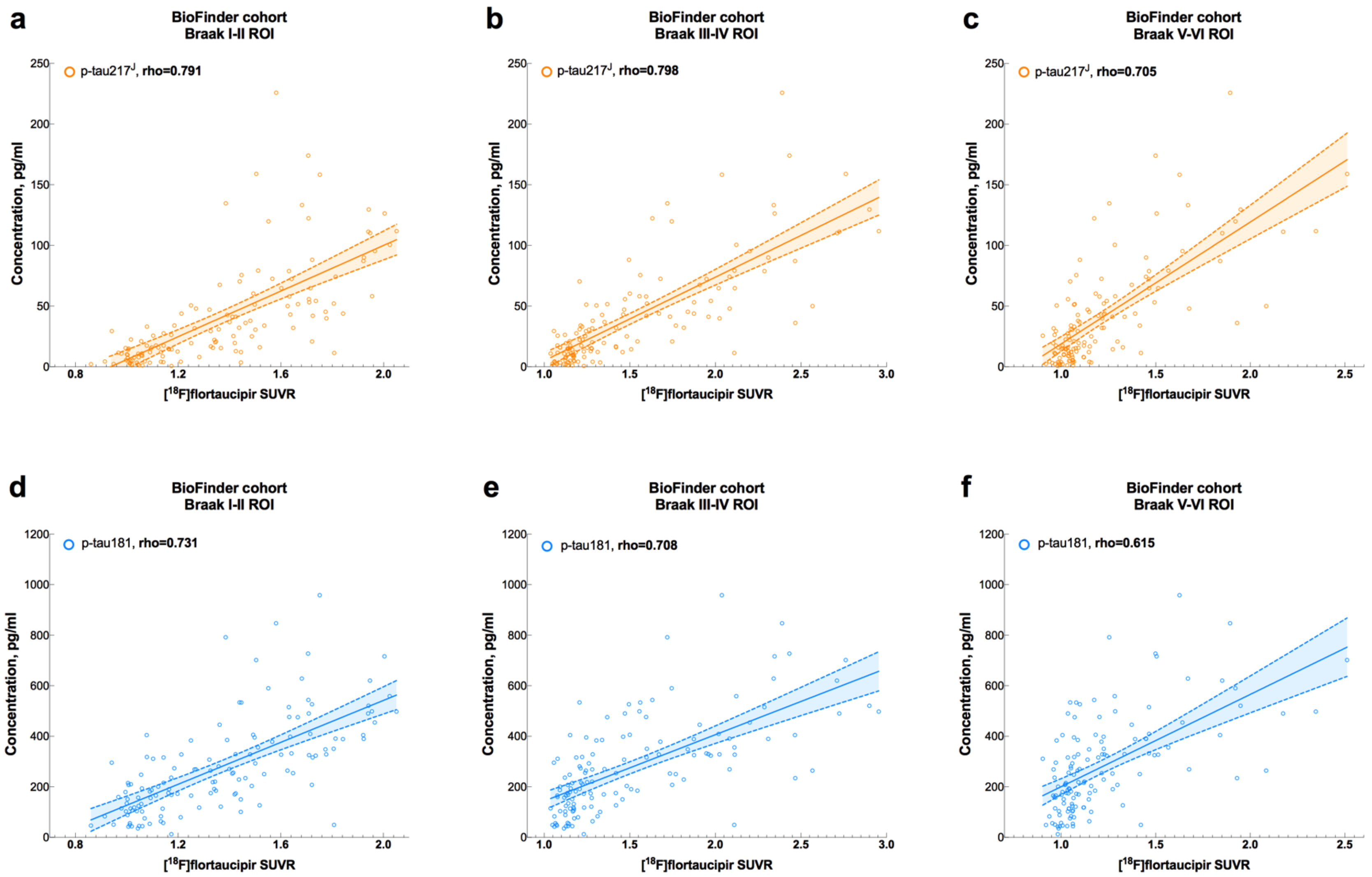

Associations between[^{18}F]flortaucipir and p-tau. BioFINDER cohort, associations between [^{18}F]flortaucipir retention in *a priori* defined brain regions linked to tau pathology in AD and CSF p-tau217^{J} (a-c) and p-tau181 (d-f) in Aβ^{+} study participants. Data are shown as Spearman correlation coefficients (rho); lines are linear regression lines with 95% CI (shaded area).

Supplementary Figure 6

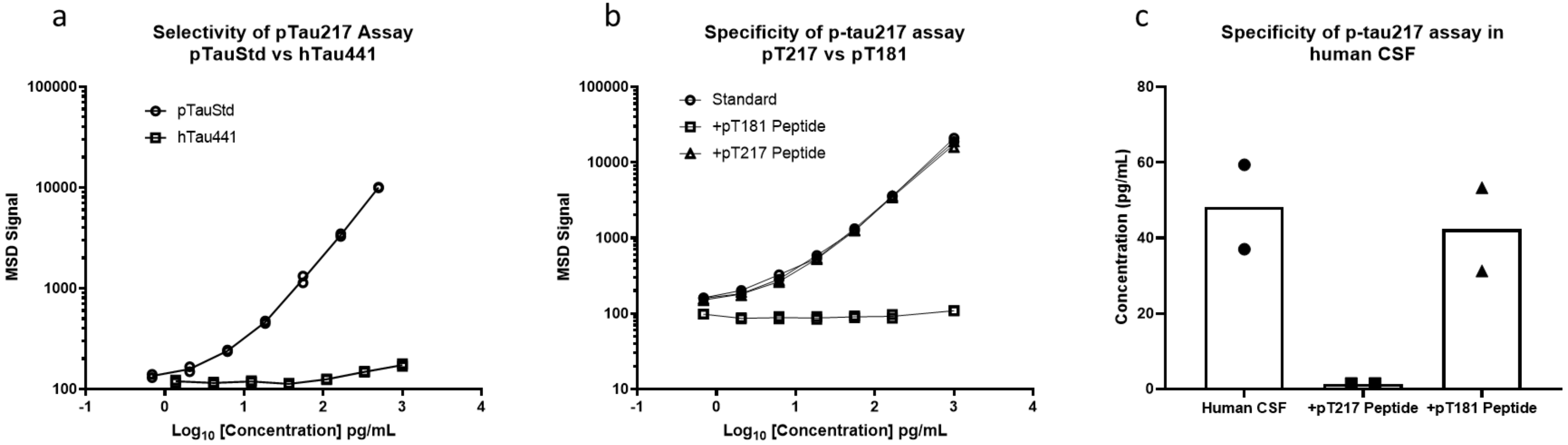

Selectivity and Specificity of p-tau217 Assay. (**a**) Selectivity is demonstrated through comparison of phosphorylated vs non-phosphorylated recombinant tau. Scatter plots are showing both replicates from the experiment and a mean connecting line is shown as visual aid only. (**b** and **c**) Specificity for the p-tau217 site was demonstrated both in assay buffer (**b,** replicates =2) and human CSF matrix (**c,** n=2). In (**c**), data are mean; both pTau217 peptide samples were below detection limit and results shown are imputed to the lower limit of quantitation.

REFERENCES

1. Rosner, B., Wang W., Eliassen H., Hibert E. Comparison of Dependent Pearson and Spearman Correlation Coefficients with and without Correction for Measurement Error. *J Biom Biostat* **6**, 1-9 (2015).

2. Robin, X., *et al.* pROC: an open-source package for R and S+ to analyze and compare ROC curves. *BMC bioinformatics* **12**, 77 (2011).

3. Mattsson, N., *et al.* Comparing (18)F-AV-1451 with CSF t-tau and p-tau for diagnosis of Alzheimer disease. *Neurology* **90**, e388-e395 (2018).
