# Supplementary material for: Cerebrospinal fluid p-tau217 performs better than p-tau181 as a biomarker of Alzheimer’s disease
Source: Nat Commun. 2020 Apr 3;11:1683. doi: 10.1038/s41467-020-15436-0 (PMC7125218; doi:10.1038/s41467-020-15436-0)
Supplement: Supplementary file 2 — Reporting Summary [file 41467_2020_15436_MOESM2_ESM.pdf]

nature research

Corresponding author(s): Oskar Hansson, Shorena Janelidze

Last updated by author(s): Mar 2, 2020

Reporting Summary

Nature Research wishes to improve the reproducibility of the work that we publish. This form provides structure for consistency and transparency in reporting. For further information on Nature Research policies, see Authors & Referees and the Editorial Policy Checklist .

Statistics

For all statistical analyses, confirm that the following items are present in the figure legend, table legend, main text, or Methods section.

n/a	Confirmed	
☐	☒	The exact sample size (*n*) for each experimental group/condition, given as a discrete number and unit of measurement
☒	☐	A statement on whether measurements were taken from distinct samples or whether the same sample was measured repeatedly
☐	☒	The statistical test(s) used AND whether they are one- or two-sided<br>*Only common tests should be described solely by name; describe more complex techniques in the Methods section.*
☐	☒	A description of all covariates tested
☐	☒	A description of any assumptions or corrections, such as tests of normality and adjustment for multiple comparisons
☐	☒	A full description of the statistical parameters including central tendency (e.g. means) or other basic estimates (e.g. regression coefficient) AND variation (e.g. standard deviation) or associated estimates of uncertainty (e.g. confidence intervals)
☐	☒	For null hypothesis testing, the test statistic (e.g. *F*, *t*, *r*) with confidence intervals, effect sizes, degrees of freedom and *P* value noted<br>*Give P values as exact values whenever suitable.*
☒	☐	For Bayesian analysis, information on the choice of priors and Markov chain Monte Carlo settings
☒	☐	For hierarchical and complex designs, identification of the appropriate level for tests and full reporting of outcomes
☒	☐	Estimates of effect sizes (e.g. Cohen's *d*, Pearson's *r*), indicating how they were calculated

Our web collection on statistics for biologists contains articles on many of the points above.

Software and code

Policy information about availability of computer code

Data collection	*Provide a description of all commercial, open source and custom code used to collect the data in this study, specifying the version used OR state that no software was used.*
Data analysis	SPSS version 24 (IBM, Armonk, NY, US), SAS (9.4), R studio and R version 3.5.3 (package pROC); FreeSurfer (version 5.3), SPM12 (http://www.fil.ion.ucl.ac.uk/spm), CARET v5.65 (Van Essen Lab; http://brainvis.wustl.edu), Prism 8.

For manuscripts utilizing custom algorithms or software that are central to the research but not yet described in published literature, software must be made available to editors/reviewers. We strongly encourage code deposition in a community repository (e.g. GitHub). See the Nature Research guidelines for submitting code & software for further information.

Data

Policy information about availability of data

All manuscripts must include a data availability statement. This statement should provide the following information, where applicable:

- Accession codes, unique identifiers, or web links for publicly available datasets
- A list of figures that have associated raw data
- A description of any restrictions on data availability

Anonymized data will be shared by request from any qualified investigator for the sole purpose of replicating procedures and results presented in the article and as long as data transfer is in agreement with EU legislation on the general data protection regulation.

Field-specific reporting

Please select the one below that is the best fit for your research. If you are not sure, read the appropriate sections before making your selection.

- [x] Life sciences
- [ ] Behavioural & social sciences
- [ ] Ecological, evolutionary & environmental sciences

For a reference copy of the document with all sections, see nature.com/documents/nr-reporting-summary-flat.pdf

Life sciences study design

All studies must disclose on these points even when the disclosure is negative.

Sample size

The study included the Swedish BioFINDER cohort (n=194) and an independent EXPEDITION3 trial validation cohort (n=32). The BioFINDER cohort was a convenience cohort and all available plasma samples were analyzed in this study. In the EXPEDITION3 trial, overall, 32 participants had chosen to undergo both flortaucipir PET and lumbar puncture at baseline (before any treatment) and all 32 participants were included in the present study. The results are convergent and positive. There is no indication that we were insufficiently powered for these analyses.

Data exclusions

No data were excluded from the analysis.

Replication

The main results in the Swedish BioFINDER cohort were validated using a different p-tau217 immunoassay and corroborated in an independent EXPEDITION3 trial cohort (n=32).

Randomization

No allocation into experimental groups were performed, therefore randomization is not relevant to this study. Statistical analyses were controlled for potential confounding effect of age and sex.

Blinding

All CSF and PET measurements were performed by individuals who were blinded to the clinical data. This is not an interventional trial and therefore blinding during data analysis was not relevant.

Reporting for specific materials, systems and methods

We require information from authors about some types of materials, experimental systems and methods used in many studies. Here, indicate whether each material, system or method listed is relevant to your study. If you are not sure if a list item applies to your research, read the appropriate section before selecting a response.

Materials & experimental systems

n/a	Involved in the study	
x		Antibodies
x		Eukaryotic cell lines
x		Palaeontology
x		Animals and other organisms
	x	Human research participants
x		Clinical data

Methods

n/a	Involved in the study	
x		ChIP-seq
x		Flow cytometry
x		MRI-based neuroimaging

Human research participants

Policy information about studies involving human research participants

Population characteristics	Detailed information is given in Table 1, Supplementary Table 4 and Methods. In short, we present results from 2 different cohorts, the Swedish BioFINDER cohort and an independent EXPEDITION3 trial validation cohort. The BioFINDER cohort included cognitively unimpaired controls (n=65) and patients with mild cognitive impairment (MCI) due to AD (n=29), AD dementia (n=43) and non-AD neurodegenerative diseases (n=57). Cohort 2 included 32 patients with mild dementia due to AD. In the BioFINDER cohort, out of 194 participants (mean (SD) age, 72 (7) years), 86 were women. In EXPEDITION3 validation cohort, out of 32 participants (mean (SD) age, 73 (8) years), 13 were women.
Recruitment	This project was done as part of the prospective Swedish BioFINDER study with participants recruited at Skåne University Hospital and the Hospital of Ängelholm, Sweden. Recruitment of patients with cognitive symptoms or neurological diseases was done at Memory clinics and Neurology clinics. The results for the patients may therefore be biased for a specialist setting. As we already state in the discussion our findings should be validated in a primary care setting. In the EXPEDITION3 trial, overall, 32 participants had chosen to undergo both flortaucipir PET and lumbar puncture at baseline (before any treatment) and all 32 participants were included in the present study. The recruitment in the EXPEDITION 3 study has been described in Honig et al. (N Engl J Med, 2018).
Ethics oversight	The BioFINDER study was approved by the Regional Ethics Committee in Lund, Sweden, and all participants gave their written informed consent to participate in the study. Samples for the validation cohort were collected as part of a phase 3 clinical trial conducted in accordance with the Declaration of Helsinki for experiments involving human research. All participants gave their informed consent to participate in the study.

Note that full information on the approval of the study protocol must also be provided in the manuscript.
